# Combining Molecular Dynamics and Machine Learning to Predict Drug Resistance Causing Variants of BRAF in Colorectal Cancer

**DOI:** 10.3390/molecules30173556

**Published:** 2025-08-30

**Authors:** Longsheng Xie, Christopher Lockhart, Dmitri K. Klimov, Mohsin Saleet Jafri

**Affiliations:** 1 School of Systems Biology, George Mason University, Fairfax, VA 22020, USA; lxie2@gmu.edu (L.X.); clockha2@gmu.edu (C.L.); dklimov@gmu.edu (D.K.K.); 2 Center for Biomedical Engineering and Technology, University of Maryland School of Medicine, Baltimore, MD 21201, USA

**Keywords:** colorectal cancer, BRAF, molecular dynamic simulation, machine learning

## Abstract

The BRAF protein regulates cell growth and division through key signaling pathways. Mutations in BRAF, particularly the V600E variant, are frequently observed in colorectal cancer (CRC) and are associated with poor prognosis and therapeutic challenges. Tumors harboring certain BRAF mutations often exhibit primary resistance to BRAF inhibitor monotherapies. Over time, these tumors can also develop acquired resistance, further complicating treatment. In this study, we employed replica exchange molecular dynamics simulations combined with machine learning techniques to investigate the structural alterations induced by BRAF mutations and their contribution to drug resistance. Our analyses revealed that conformational changes in mutant BRAF proteins associated with dabrafenib residues psi494, phi600, phi644, phi663, psi675, and phi677 were sufficient for classifying drug-resistant vs. drug-sensitive variants. Similarly, for vemurafenib, residues psi450, phi484, phi495, phi518, psi622, and phi622 were the key residues that influence drug binding and resistance mechanisms. These residues are located in the N-lobe of CR3, which is responsible for ATP binding and the regulation of BRAF kinase activity. These findings offer deeper insights into the molecular basis of BRAF-driven resistance and provide predictive models for phenotypic outcomes of various BRAF mutations. The study underscores the importance of targeting specific BRAF variants for more effective, personalized therapeutic strategies in drug-resistant CRC patients.

## 1. Introduction

### 1.1. Colorectal Cancer

Colorectal cancer (CRC) is a major global health concern and is the third most commonly diagnosed cancer in both men and women worldwide. It accounts for a significant proportion of cancer-related deaths, ranking as the second leading cause of cancer mortality [1]. In 2024 the American Cancer Society estimated that 53,010 deaths were due to colorectal cancer in the United States, highlighting the deadly nature of the disease [2]. Despite advancements in screening and treatment, CRC remains a leading cause of cancer-related mortality [3]. Alarmingly, about 90% of these cancer deaths are attributed to drug resistance, a major barrier to effective cancer treatment [4,5]. Drug resistance typically arises from genetic mutations in key genes involved in cell growth, division, and apoptosis, rendering standard treatments less effective and complicating disease management.

CRC represents the most common form of gastrointestinal cancer and is responsible for a significant burden on healthcare systems. Several factors contribute to the risk of developing colorectal cancer, including poor dietary habits, smoking, chronic intestinal inflammation, the presence of polyps, hereditary predispositions, and advancing age [2]. Notably, over 90% of patients diagnosed with CRC are over the age of 50, with the median age at diagnosis being 64 [2]. However, it is worth noting that in younger patients, the disease tends to present in a more aggressive form, posing additional challenges for treatment. Early detection, through screening methods such as colonoscopy, has proven crucial in improving survival rates, yet many cases are diagnosed at advanced stages when therapeutic options are limited.

The role of genetic mutations, particularly those affecting oncogenes like BRAF, and the development of drug resistance continue to be central challenges in colorectal cancer treatment [6]. Addressing the molecular underpinnings of these resistance mechanisms through targeted therapies and personalized medicine holds the promise of improving outcomes for CRC patients, particularly those who develop resistance to standard chemotherapy and targeted therapies. By understanding the factors that drive the progression of colorectal cancer and the emergence of drug resistance, researchers and clinicians can work towards developing more effective treatment strategies that can prolong survival and improve the quality of life for patients afflicted with this devastating disease.

### 1.2. Variants of Unclassified Significance

Colorectal cancer is commonly treated with chemotherapy, but when certain genetic variations, such as BRAF mutations, are present, targeted therapies are incorporated into the treatment strategy. Genetic alterations in BRAF are common in colorectal cancer, alongside others like KRAS, which predicts resistance to epidermal growth factor receptor (EGFR) monoclonal antibodies [7]. Given the critical role of BRAF in drug resistance and cancer progression, it remains a key focus in studies exploring targeted treatments for colorectal cancer. However, actionable BRAF mutations account for only a small fraction of the genetic landscape in colorectal cancer. Many mutations, including BRAF variants of uncertain significance (VUS), remain poorly understood and may contribute to drug resistance in patients [6,8]. Consequently, valuable genetic information is often underutilized in guiding treatment protocols.

### 1.3. BRAF Structure and Function

BRAF has three domains, or conserved regions: CR1, CR2, and CR3. The CR1 domain (residues 155–280) contains the Ras-binding domain that initially binds Ras-GTP and is responsible for autoinhibition of BRAF. The CR2 domain is a flexible linker between the CR1 and CR3 domains and contains the phosphorylation site for 14-3-3 proteins (residues ~280–457). The CR3 domain (residues 458–717) is considered to be the kinase domain and contains the ATP-binding site (residues 483 and 501—salt bridge; 529—gatekeeper residue; 505, 514, 593, 594, and 595—Raf specificity pocket), the P-loop, which stabilizes the non-transferable phosphate groups of ATP (residues 458–475), the catalytic loop (residues 574–581) and the activation loop (residues 593–600). BRAF inhibitors dabrafenib and vemurafenib bind near the ATP-binding site. It is important to note that the variants that are known to be involved in cancer and drug resistance are found in the CR3 domain, where they can exert the effect of constitutive activation of BRAF kinase activity, which would promote growth and proliferation.

### 1.4. Molecular Dynamic Simulation

Structural changes caused by genetic mutations can be analyzed through simulations of variant protein structures. Computational biology methods, such as Replica Exchange with Solute Tempering (REST2), are employed to gain a deep understanding of the complex behavior of each variant in aqueous solutions [9]. Recent advancements in molecular computing algorithms and the powerful capabilities of graphics processing units (GPUs) enable the simultaneous performance of many random calculations over extended periods, allowing for comprehensive analysis [10].

Molecular dynamics simulations, using REST2, provide insights into protein conformations by determining the distribution of structures at equilibrium [11]. REST2 specifically modulates only the protein component of the system, optimizing computational efficiency by reducing the number of replicas needed without sacrificing sampling accuracy. By focusing on the protein rather than the solvent, REST2 enables more efficient simulations compared to traditional methods like Replica Exchange Molecular Dynamics (REMD) [12]. This study will use a well-tested explicit solvent model to further enhance the accuracy of computational predictions, improving upon the implicit solvent approach used in previous analyses [12].

## 2. Results

The primary goal of this research is to identify the key residues of the BRAF protein that contribute to drug resistance in various clinically significant variants. Our approach is based on the hypothesis that specific local structural changes in BRAF variants, particularly in regions relevant to drug binding, are associated with their resistance to inhibitors like dabrafenib and vemurafenib. By analyzing the conformational dynamics of BRAF through REST2 simulations, we aim to uncover the structural features that differentiate drug-resistant variants from sensitive ones. These insights can help elucidate how certain BRAF mutations influence drug binding and resistance mechanisms, contributing to the development of more effective targeted therapies.

### 2.1. REST2 Simulations of BRAF

REST2 simulations were employed to explore the conformational ensembles of 16 clinically significant BRAF variants, including the wild type. An ensemble of structures was gathered from the final 1000 frames across all three trajectories, resulting in 3000 structures for each variant. Each simulation was run for 20 ns to ensure the system reached equilibrium (Figure S1, Supplementary Materials).

**Figure 1:**
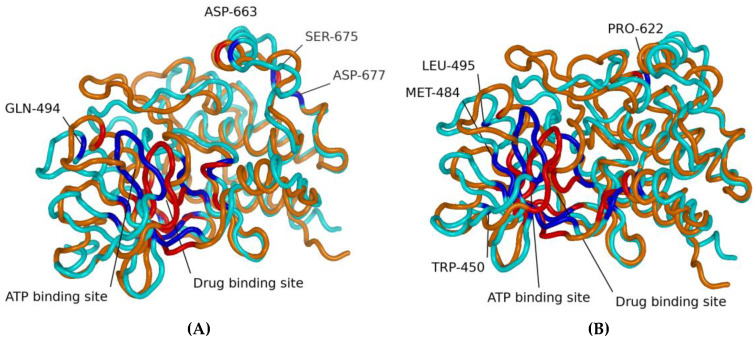
Molecular structures obtained from REST2 molecular dynamics simulations. Each panel shows selected residues for the dabrafenib and ATP binding sites (**A**) and for the vemurafenib and ATP binding sites (**B**). In all figures, cyan represents the WT, and orange represents the G469A mutant. The blue represents residues identified by machine learning feature selection as important for differentiating sensitive from resistant to the WT, and the red residues identified by machine learning feature selection as important for differentiating sensitive from resistant to the G469A mutant.

Figure 1 shows the molecular structures obtained from REST2 molecular dynamics simulations. Figure 1A shows the dabrafenib and ATP binding sites in BRAF. Figure 1B shows the vemurafenib and ATP binding sites. While the two sites have some overlapping residues, the lines point to the approximate center of each site. It is easy to note the differences between the drug-sensitive WT and drug-resistant G469A variants. It is also important to note that while the WT binds the drugs dabrafenib and vemurafenib, application of the drug does not suppress growth and proliferation due to paradoxical stimulation of the MAPK pathway [13,14].

### 2.2. Machine Learning Analysis Results

Machine learning was applied to identify key dihedral angles (phi and psi) from REST2 simulations that characterize the drug resistance of BRAF variants to dabrafenib and vemurafenib. In total, 3000 structures were analyzed for each of the 12 dabrafenib and 11 vemurafenib known drug-resistant variants, and the corresponding phi and psi angles were extracted.

Decision trees were used to determine which dihedral angles could be used to classify these variants, as shown in the figures below. Once the first tree determined the classifying angles, those angles were then removed from the feature set. A decision boundary is decided by testing all the possible decision boundaries splitting the dataset and choosing the one that minimizes the Gini impurity of the two splits. Moreover, Gini impurity is a metric that measures the probability of a randomly chosen element (here, a variant) being incorrectly classified, i.e., the probability of choosing the element times the probability of being misclassified. Samples describe the percentage of variants that need to be classified.

For dabrafenib, three decision trees were generated (Figure S2, Supplementary Materials). In the first tree, phi664 and phi600 were identified as key angles, enabling a clear separation of resistant and sensitive variants. In the second tree, phi663 and psi494 were found to be important features, and in the third tree, psi675 and phi677 played a crucial role in classification. The classification accuracy for these decision trees was 100%, with Gini impurity values minimized across the dataset splits. Each tree tested potential decision boundaries and selected the one that resulted in the most accurate classification of variants. For vemurafenib, four decision trees were generated (Figure S3, Supplementary Materials). Key angles identified in the first tree were psi622. Subsequent trees identified additional angles, such as phi484, psi450, phi495, phi518, and phi622, as important contributors to classifying resistance.

**Figure 2:**
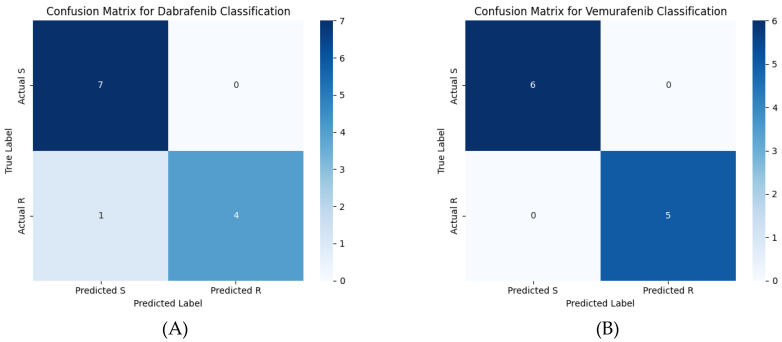
Confusion matrices for (**A**) the dabrafenib machine learning model and (**B**) the vemurafenib machine learning model. The scale bar color indicates the number of variants in each quadrant of the confusion matrix.

Our machine learning model was applied to predict the resistance of BRAF variants to both dabrafenib and vemurafenib. For dabrafenib, the model achieved an accuracy of 91.67% (Table 1). In the first column, the table shows the phi and psi dihedral angles that were used as the features in the random forest machine learning model. The second column shows the decision trees in Figure 2 that determined these dihedral angles. The third column shows the accuracy of each model. It accurately predicted the sensitivity of key variants, including V600E, V600M, and V600K, as well as the resistance of variants such as L505H and G466E. However, a small deviation in prediction accuracy was observed for variant K601E, indicating the need for further analysis of these specific cases. The model used decision tree 1 (phi644 and phi600), decision tree 2 (phi663 and psi494), and decision tree 3 (psi675 and phi677). Notably, the model with decision trees 1, 2, and 3 produced the same accuracy and predictions as the model with only decision trees 2 and 3, suggesting that decision tree 1 may not significantly contribute to the overall prediction accuracy in this context.

For vemurafenib, the model demonstrated exceptional performance in predicting the sensitivity (S) or resistance (R) of variants to vemurafenib, achieving 100% accuracy (Table 2). In the first column, the table shows the phi and psi dihedral angles that were used as the features in the random forest machine learning model. The second column shows the decision trees in Figure S3 that determined these dihedral angles. The third column shows the accuracy of each model. It accurately classified well-known variants, including V600E, V600M, and L597R, as either sensitive or resistant. Additionally, the model successfully predicted the behavior of other variants, such as S467V, G469V, and K601E. When comparing different combinations of decision trees, we observed that the model including decision trees 1, 2, 3, and 4 produced the same prediction as the model with decision trees 1, 2, and 3, indicating that decision tree 4 did not contribute additional predictive power. Similarly, the model including decision trees 1, 3, and 4 gave the same prediction as the model with decision trees 1 and 3, suggesting that decision tree 2 was essential for distinguishing certain variants. Based on these observations, we chose the model that included decision trees 1, 2, and 3, as it provided consistent and reliable predictions. This approach ensured the robustness and generalizability of the model, making it more accurate for predicting sensitivity and resistance in a broad range of variants.

The predictions of the machine learning model using these features are shown in the confusion matrix in Figure 2. Table 3 contains the performance metrics for both the dabrafenib and vemurafenib machine learning models. The variant-by-variant predictions are summarized in Table 4 and Table 5. Table 4 describes the known drug resistance status for the BRAF variants to dabrafenib at the top. Note that the K601E variant was the only variant incorrectly classified by the machine learning model, yielding a 91.67% accuracy of the model. At the bottom, drug susceptibility/resistance predictions are made for the variants of unknown significance. Table 5 describes the known drug resistance status for the BRAF variants to vemurafenib at the top. Note that all the known variants were correctly classified, yielding 100% accuracy of the model. At the bottom, drug susceptibility/resistance predictions are made for the variants of unknown significance. Table 6 shows the predictions of other methods for comparison to the machine learning model presented in this work. AlphaMissense predicts all the variants to be likely pathogenic, most likely due to their role in cancer, but makes no predictions about their drug resistance status. PredictSNP predicts pathogenicity for all the variants except V600M but also does not give any predictions about drug resistance status.

**Figure 3:**
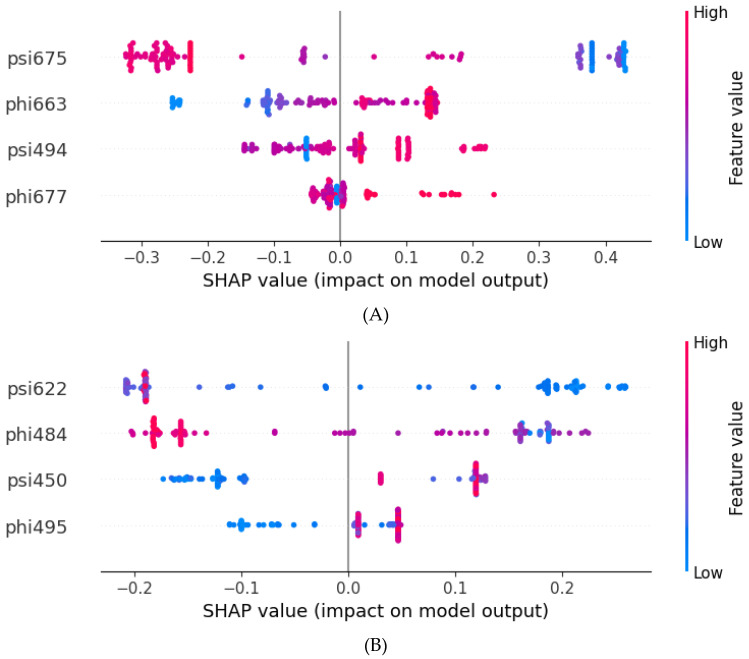
Feature importance evaluated by SHAP values. (**A**) Dabrafenib SHAP values. (**B**) Vemurafenib SHAP values. Red indicates a higher feature value. Blue indicates a lower feature value. Positive SHAP values indicate resistance, and negative values indicate sensitivity.

In Figure 3, the *X*-axis shows the SHAP values (SHapley Additive exPlanations) that indicate how strongly each feature pushes the model’s prediction toward a particular class. Positive SHAP values mean the feature drives the prediction toward the “Resistant” class (label = 1). Negative SHAP values mean the feature drives the prediction toward the “Sensitive” class (label = 0). The magnitude of the SHAP value reflects the strength of this contribution. In Figure 3, red indicates a higher feature value, while blue indicates a lower feature value. There are several pieces of information that can be inferred from the plot. For psi675, the higher values (red dots) strongly drive predictions toward “Sensitive” (negative SHAP), and the lower values (blue dots) strongly drive predictions toward “Resistant” (positive SHAP). For phi663, the lower values (blue) tend toward “Sensitive”, and the higher values (red) tend toward “Resistant”. Dihedral angles psi494 and phi677 display mixed patterns but still demonstrate clear and consistent contributions. The current SHAP analysis clearly demonstrates stable and consistent feature importance and reflects the biological and structural relevance of selected features.

## 3. Discussion

This study provides significant insights into the structural dynamics of BRAF variants and their relationship to drug resistance in colorectal cancer, using molecular dynamics simulations and machine learning techniques. REST2 simulations generated high-resolution structural data, revealing key dihedral angles in the BRAF protein variants that correlate with resistance to dabrafenib and vemurafenib. The application of machine learning, particularly the random forest algorithm, allowed us to identify critical features from the structural data, which were instrumental in distinguishing between drug-resistant and sensitive variants.

### 3.1. Structure–Function Insights

**Figure 4:**
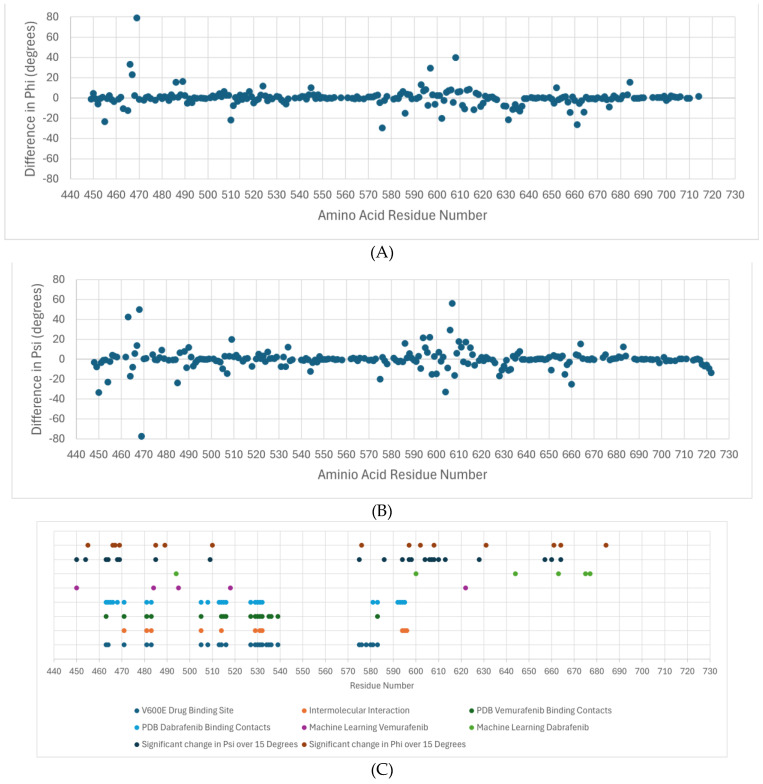
Residues with differences in the average dihedral angle between drug-resistant and susceptible for each residue. (**A**) The average difference in phi dihedral angle between sensitive and resistant for the dabrafenib study. (**B**) The average difference in psi dihedral angle between sensitive and resistant for the dabrafenib study. (**C**) A summary of the important residues for drug binding based on the machine learning results (ML), drug binding site, reported intermolecular interactions between BRAF and ligand, contact residues between BRAF and ligand, and analysis of molecular simulation results.

**Figure 5:**
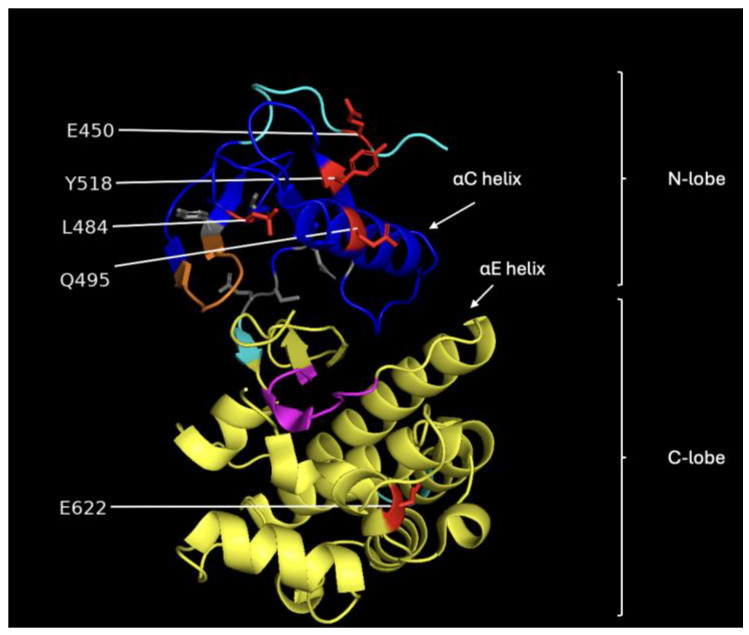
Image showing the structural features of the CR3 domain of BRAF. The blue region represents the N-lobe, and the yellow region corresponds to the C-lobe. The orange segment is the P-loop, magenta marks the catalytic loop, and the gray residues indicate the hydrophobic pocket. The red residues are the ones we selected for classifying vemurafenib sensitivity.

Figure 4A shows the differences in the average phi dihedral angle between drug-resistant and susceptible for each residue in the dabrafenib study. The average difference in psi dihedral angle between sensitive and resistant for the dabrafenib study (Figure 4B). Note that large differences are found between residues 450–470 and 575–635, with smaller regional differences around 510 and around 660. Previous studies have identified that the active site of BRAF variant V600E for vemurafenib and other related drugs consists of residues Ile-463, Gly-464, and Val-471 of the P-loop; Leu-505 and Thr-508 of the αC-helix; Arg-575, Asp-576, Lys-578, Asn-580, and Asn-581 of the catalytic site; Ile-527, Thr-529, Gln-530, Trp-531, Cys-532, Gly-534, Ser-535, Ser-536 and His-539 of the N-lobe; and Phe-583, Gly-593, Glu-600, Lys-601, Arg-603, Tpr-604, and Ser-605 of the C-lobe [17]. These are shown in Figure 4C in blue. The residues that participate in the intermolecular interaction of the drug and BRAF are Val-471, Ala-481, Lys-483, Leu-505, Leu-514, Thr-529, Trp-531, Cys-532, Asp-594, Phe-595, and Gly-596 and are deemed essential for the activity of these class inhibitors are shown in orange [17]. The PDB structure 4RZV of the vemurafenib drug binding site shows residues 463, 471, 472, 481, 482, 483, 505, 514-516, 527, 529–532, 535, 536, 539, 583, and 593–596 as the binding site (dark green). In PDB structure 5CSW, the dabrafenib drug binding site is 463–466, 468, 471, 481, 483, 505, 508, 513–516, 527, 529–532, 580, 581, 583, and 592–595 (light blue). They are all part of the CR3 domain, which is the kinase domain. The feature residues 484 and 518 lie adjacent to contact residues in the drug binding site, and 450 and 495 are close enough to likely affect drug binding, as shown in Figure 5.

Machine learning applied to the dihedral angle data as features found that residues 450, 484, 495, 518, and 622 (purple) were sufficient to classify vemurafenib resistance/sensitivity of variants. Similarly, residues 494, 600, 644, 663, 675, and 677 (light green) were sufficient to characterize the dabrafenib response. Finally, statistical evaluation of significant differences between the average dihedral angle value between the sensitive variants and the resistant variants using the t-test yielded many that were statistically significant. In our study we chose those with more than a 15-degree difference, which corresponds to the standard deviation over residues in wild-type BRAF. For phi, the residues are 455, 466, 467, 469, 486, 489, 510, 576, 597, 602, 608, 631, 661, and 684 (dark blue) and for phi the residues are 450 454, 463, 464, 468, 469, 485, 509, 575, 586, 594, 597, 598, 604, 606, 607, 608, 610, 613, 628, 657, 660, and 664 (brown).

A comparison between these sets of residues shows that the machine learning and statistical analysis of the molecular simulation identified many residues around the binding sites and suggests that these are very important for understanding drug resistance. The residues found by the machine learning lie in the CR3 domain except for one in the CR2 domain phosphorylation site for 14-3-3 proteins (residues ~280–457). Interestingly, there were several residues in the range of 620–685 that were not previously implicated as important for binding. This region is part of the kinase domain (CR3) that is responsible for the catalytic activity of the BRAF protein, which is to phosphorylate and activate downstream proteins in the RAS/MAPK signaling pathway [18]. Figure 5 shows an image of the BRAF CR3 domain with the different functional regions identified. Most of the amino acid residues identified by the machine learning approach are in the N-lobe of CR3, which is responsible for ATP binding and the regulation of BRAF kinase activity [19].

The model also correctly identified several sensitive variants, including V600E and L597R. These results confirm that the structural variations observed through REST2 simulations can be effectively used to predict drug response. This model demonstrated the robustness of our approach, where decision trees identified significant dihedral angles, such as M484, W450, L495, and P622, that played crucial roles in determining variant resistance for vemurafenib. However, for dabrafenib, the decision trees, particularly those focusing on dihedral angles such as Q494, D663, S675, and D677, were instrumental in the classification process and provided strong predictive power for the majority of variants. The residues identified with machine learning as important for determining drug resistance either overlap or are adjacent to those identified in the scientific literature.

### 3.2. Machine Learning Prediction of Drug Resistant Variants

For vemurafenib, the machine learning model achieved 100% accuracy, correctly predicting the resistance or sensitivity of variants, including well-known resistant mutations such as G469A and V600E+L505H. In contrast, for dabrafenib, the model achieved an accuracy of 91.67%. While the model correctly classified the majority of resistant and sensitive variants, a slight deviation was observed in predicting the resistance status of the K601E variant. This suggests that further refinement may be necessary to improve the model’s prediction accuracy for certain mutations.

In this study, we chose to use the clinically relevant classifications of resistant and sensitive. The experimental data on the IC50 of variants is limited and has been shown to be inconsistent with clinical findings. Table 6 shows the variants with measured IC50 values for dabrafenib. There is a clear separation into two classes: <5 nm and >1 µM. This justifies the use of two classes. For example, the variants L597V (susceptible clinically to dabrafenib) and G469A (resistant clinically to dabrafenib) both showed an IC50 of (>1 µM) in cell lines [16]. We therefore choose to rely on the clinical classification.

There have been other methods developed to predict the pathogenicity of variants. These include the online servers such as AlphaMissense and PredictSNP that are shown in Table 7. These predict the variants as likely pathogenic or deleterious but are not made to specifically address drug resistance or sensitivity. Other methods have been developed to predict the functional consequences of variants. In one such study, an XGBoost-based machine used position, frequency, consequence for the canonical *BRCA2* transcript, and deleteriousness prediction scores from several tools as features to obtain high accuracy prediction of BRCA variants [20]. The software 3Cnet uses recurrent neural networks to analyze the amino acid context of human variants to predict pathogenicity [21]. Another study used gene-specific machine learning rather than disease-specific to predict the pathogenicity of BRCA variants [22]. Deep learning has also been applied to predict pathogenicity in the Missense Variant Pathogenicity prediction method for variants in hereditary cancer [23]. However, none of these methods specifically address the question of drug resistance/sensitivity of variants.

In this study, a few features (dihedral angles) were selected and yielded better results than using all the dihedral angles. In machine learning, feature selection can lead to an improvement of model results. In a model with a large feature set, some of the features might not correlate well with the different classes. For example, there might be a feature that changes little across classes or one whose large variance appears as “noise”. By eliminating such features and only keeping those with relevant information content, the machine learning model can have better performance with the selected set of features over regular parameters.

There have been previous studies applying machine learning to features from molecular simulations from our research group and others. In one study we used macroscopic measures such as the twist and length of molecular features in calmodulin and the dihedral angles to differentiate between different diseases caused by different variants in the same protein [24]. We have also made predictions about Venetoclax resistance of BCL-2 variants [25]. Tam and co-workers applied deep learning to Ramachandran plots of the dihedral angles to functionally classify genetic variants of proteins [26].

In conclusion, the combination of molecular dynamics and machine learning is a powerful tool to understand how changes in protein structure caused by genetic variants can lead to changes in function. This method can be leveraged to develop a classification model for predicting drug resistance/sensitivity for previously unclassified variants. These predictions could be used to determine which variants should be the subject of further experimental and clinical studies.

## 4. Materials and Methods

**Figure 6:**
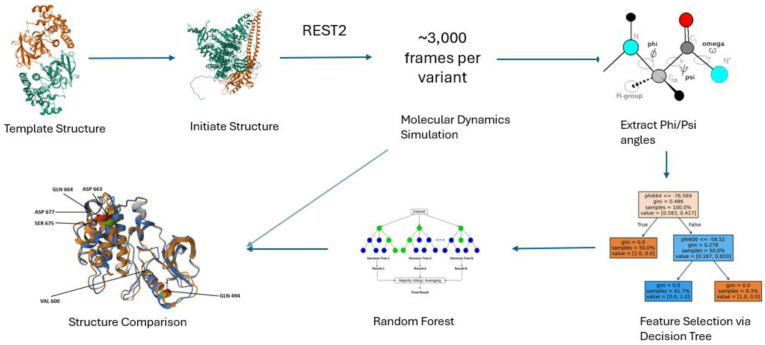
Overview of the simulation-based machine learning framework. Starting from template protein structures, point mutations are modeled to generate variant structures. Molecular dynamics simulations using REST2 are run in three independent trajectories per variant, each producing 10,000 frames. From each trajectory, the final 1000 frames are extracted, resulting in a total of ~3000 frames per variant. Backbone dihedral angles are computed from these frames and filtered via decision tree–based feature selection. Selected features are used to train a random forest classifier to predict whether a mutation confers drug resistance. Structural comparison of average conformations provides additional mechanistic insight into resistance-associated changes.

The workflow of this computational study is shown in Figure 6. It consists of obtaining a template structure, obtaining an initial structure, performing REST2 molecular dynamics simulation, extracting the phi and psi dihedral angles, performing feature selection using classification trees, building a random forest machine learning model to classify variants, and structural analysis.

### 4.1. BRAF Structure

The PDB X-ray crystal structure for BRAF (1UWH) with the ligand removed was used as the starting structure for the wild-type BRAF simulations. BRAF variants were selected based on data from the CIViC (Clinical Interpretation of Variants in Cancer) and JAX (Jackson Labs) cancer variant knowledgebases for REST2 simulations. These variants include key mutations relevant to drug resistance, and their sequences are provided in Table 8. For each variant, we generated a variant structure from the wild-type template, solvated and ionized the system, and then carried out the standard protocol: energy minimization, heating, and equilibration before production and REST2 runs.

### 4.2. REST2 Simulations

In these studies, a single BRAF peptide (with no ligand) in water was modeled using the CHARMM36m force field, with acetylated N-termini and amidated C-termini capping the peptide [27]. Each system was modeled in the NPT ensemble, and we employed the CHARMM36m force field along with the TIP3P water model and neutralized with counterions to explicitly simulate the hydration environment [27,28,29]. The use of REST2 with explicit solvent provides a more realistic representation of BRAF’s structural behavior compared to implicit solvent models. Molecular dynamics simulations were performed using NAMD [30], following the established parameters used in previous studies by our group [24,31]. The simulations employed a 1 fs integration step, with full electrostatic interactions and van der Waals forces smoothly switched off between 8 and 12 Å. Temperature was controlled using underdamped Langevin dynamics, with a damping coefficient of 5 ps^−1^, and pressure was maintained at 1 atm using the Langevin piston method. The main goal of the REST simulations was to produce a collection of states that correctly adhere to the Boltzmann distribution. The Langevin thermostat was chosen because it rigorously reproduces Boltzmann sampling. The Langevin damping coefficient used in our simulations is 5 ps^−1^, and it corresponds to the underdamped regime, which facilitates structural transitions and “speeds up” dynamic properties. The REST algorithm is not designed to generate correct kinetics but to sample state probabilities. This becomes possible because kinetic energy terms cancel out from the computation of thermodynamic averages and play no role in REST exchanges [9]. It is also worth mentioning that “incorrect” kinetics is used not only in replica exchange but also in hydrogen mass repartitioning or with “light” water simulations [32]. In all these cases, the correct Boltzmann distribution is collected, whereas kinetic properties may or may not be reproduced. This does not represent an issue, because the former but not the latter is the purpose of our studies.

Covalent bonds involving hydrogen atoms were constrained using the SHAKE algorithm, ensuring stability during the simulations, while long-range van der Waals and electrostatic interactions were computed every 2 and 4 timesteps, respectively. Long-range electrostatic interactions were computed using Particle Mesh Ewald summation, which is the standard approach in modern molecular dynamics simulations. The switching function controlling the onset of Ewald summation was defined between 8 and 12 Å. Electrostatic interactions were calculated using the Ewald summation method, and pressure was maintained at 1 atm using the Langevin piston method. These simulations aim to investigate the structural impact of BRAF variants on drug binding and resistance to inhibitors like dabrafenib and vemurafenib. This computational setup ensures the accurate simulation of BRAF variants under realistic physiological conditions, providing critical insights into the structural changes that contribute to drug resistance.

REST2 simulations were conducted using the replica exchange TCL scripts provided with NAMD (https://www.ks.uiuc.edu/Research/namd/ last accessed 5 August 2022). For the BRAF simulations, a total of R=6 replicas were utilized for each of the 16 clinically significant BRAF variants, including the wild type. These variants were selected from cancer resistance databases, such as JAX and CIViC. The temperatures were exponentially distributed in the range from 310 K to 340 K, based on the folding temperature of BRAF as predicted from [33]. Replica exchanges were attempted every 2 ps, and the simulations were run for 20 ns per replica to ensure thorough conformational sampling. Three trajectories were produced by each simulation system. Exchange acceptance rates are attached (~20% on average), which is within the recommended range for effective REST2 sampling. REST2 simulations were performed on Exxact Valence workstations with a 64-core AMD Threadripper 3995WX and 4 NVIDIA RTX A6000 GPUs.

This allowed the system to explore a broad conformational space without bias. The acceptance rate for replica exchanges across the REST2 simulations averaged 25%, ensuring efficient exchanges between replicas. As seen in Figure S1, the fluctuations in the protein structure began to stabilize after approximately 6000 frames, indicating the convergence of the simulations.

### 4.3. Feature Extraction from REST2 Trajectories to Form the Data Matrix

In this study, REST2 simulations were carried out for 15 clinically significant BRAF variants and wild type to examine their structural dynamics. For dabrafenib, we analyzed 12 known drug resistance variants, resulting in a data matrix of 36,000 rows × 552 columns. Similarly, for vemurafenib, 11 known drug resistance variants were studied, producing a 33,000 × 552 matrix. For both cases, 3000 structures were selected from the final 1000 frames of all three REST2 trajectories for each variant, as explained below in Section 4.5.

Each matrix consists of 550 columns for the phi and psi dihedral angles of the protein backbone, along with columns for the drug resistance status and variant label. The dihedral angles were extracted from the structures using Visual Molecular Dynamics (VMD), providing a detailed overview of conformational sampling for each BRAF variant. These REST2 simulations enabled us to capture the high-resolution structural changes in BRAF variants, offering insights into their structural behavior in response to drug interactions. The machine learning algorithm was consequently performed on the matrix of the resulting average of the angles from the analysis.

The TCL scripts used to extract the phi and psi angles are available on the George Mason University Dataverse at https://doi.org/10.13021/orc2020/OW4T3K (retrieved on 26 January 2022) or in the Supplemental Material. This extensive dataset allows for a thorough analysis of the structural variations in BRAF and their potential impact on drug binding and resistance mechanisms.

### 4.4. Machine Learning Analysis of BRAF

The random forest algorithm was employed to predict the classification of each BRAF variant based on their structural data. Random forest and decision tree were implemented using the RandomForestClassifier and the DecisionTreeClassifier modules from Scikit-learn version 1.6.1 in Python Relaese 3.11.12 [34]. Random forest is a robust classification algorithm that utilizes an ensemble of decision trees to distinguish data classes within the training dataset [35]. Each tree in the forest votes on the classification of a data entry, and the majority output determines the final classification. In this study, the random forest algorithm was used to classify BRAF variants into drug-resistant or drug-sensitive categories for dabrafenib and vemurafenib.

A leave-one-variant-out classification methodology was applied for testing and training. For each iteration, one BRAF variant was removed from the dataset and used as the testing variant, ensuring no sampling or training occurred on the test data. The random forest was trained using the dihedral angles (phi and psi) of the remaining variants’ structures. Once trained, the algorithm was tested on the excluded variant to predict its drug resistance classification. This process was repeated for each BRAF variant to ensure comprehensive testing across all variants in the study.

### 4.5. Simulation Convergence

There were three criteria that determined that the length of the simulation was adequate: convergence of RMSD (Figure S1), convergence of machine learning, and convergence of Ramachandran plots. To ensure that the molecular simulations of BRAF variants were converged, we first assessed the root-mean-square deviation (RMSD) of each variant. The simulations were considered converged once the RMSD values leveled off, indicating structural stability (Figure S1). Following this, machine learning and FATCAT analyses were performed on frames 9400–9599, 9600–9799, and 9800–9999 from each trajectory at 310 K [36]. The results from these subsets of 200 frames were compared to those from the last 1000 frames.

As another test for simulation convergence, Ramachandra plots were created for the last 1000 frames and compared to the penultimate 1000 frames (2000-1000 from the simulation end). The two time periods show little change in the phi and psi angle distribution, as shown in the Supplemental File rama.zip.

The machine learning results identified similar protein regions with only slight differences in specific residues when analyzing the smaller sets of 200 frames versus the full set of 1000 frames. In contrast, using the first 200 frames produced more numerous and different dihedral angles, suggesting that these frames represented non-equilibrated sampling. This analysis confirms that the dataset from each trajectory at 310 K reflects equilibrated structural conformations, providing a reliable foundation for further analyses.

## 5. Conclusions

This study successfully combined molecular dynamics simulations with machine learning to predict the drug resistance of BRAF variants to dabrafenib and vemurafenib in colorectal cancer. The results indicate that specific dihedral angles within BRAF variants can serve as reliable predictors for drug sensitivity or resistance, and decision trees were crucial in identifying these structural features. The model achieved 100% accuracy for vemurafenib predictions and 91.67% accuracy for dabrafenib predictions, demonstrating the efficacy of our approach.

Future work should focus on refining the model to further improve accuracy, particularly for challenging variants such as K601E. Additionally, expanding the dataset to include more variants of uncertain significance (VUS) could help generalize the findings and improve the prediction of drug resistance across a broader spectrum of BRAF mutations. These insights could guide the development of personalized treatment strategies, improving outcomes for patients with drug-resistant colorectal cancer.

## Figures and Tables

**Table 1:** Random forests features and accuracy for dabrafenib.

Features	Decision Tree	Accuracy
psi494, phi600, phi644, phi663 psi675, phi677	1, 2, 3	91.67%
psi494, phi663, psi675, phi677	2, 3	91.67%
phi600, phi644, psi675, phi677	1, 3	75.00%
phi600, phi644, phi663 psi494	1, 2	66.67%

Note: The decision tree numbers refer to the decision trees in Figure S2.

**Table 2:** Random forests features and accuracy for vemurafenib.

Features	Decision Tree	Accuracy
psi450, phi484, phi495, phi518, phi622, psi622	1, 2, 3, 4	100.00%
psi450, phi484, phi495, psi622	1, 2, 3	100.00%
psi450, phi495, phi518, phi622 psi622,	1, 3, 4	100.00%
psi450, phi495, psi622	1, 3	100.00%
phi484, phi518, phi622, psi622	1, 2, 4	75.00%
psi450, phi495, phi518, phi622	3, 4	66.67%
psi450, phi484, phi495, phi518, phi622	2, 3, 4	81.82%

Note: The decision tree numbers refer to the decision trees in Figure S3.

**Table 3:** Machine learning model performance metrics.

Metric	Dabrafenib	Vemurafenib
Accuracy	0.92	1.00
Specificity	1.00	1.00
Matthews Correlation Coefficient (MCC)	0.84	1.00
**Sensitive**		
Sensitivity (Recall)	1.00	1.00
Precision	0.88	1.00
F1-Score	0.93	1.00
**Resistant**		
Sensitivity (Recall)	1.00	1.00
Precision	0.80	1.00
F1-Score	0.89	1.00

**Table 4:** Dabrafenib BRAF variants resistance prediction.

Variants with Known Classification		
Variant Substitution	Dabrafenib	Prediction Dabrafenib
V600E	S	S
V600M	S	S
V600K	S	S
V600D	S	S
V600R	S	S
G466E	R	R
WT	S	S
L597S	S	S
K601E	R	S
G469A	R	R
G469V	R	R
S467L	R	R
		Accuracy = 91.67%
**Variants of Unknown Significance**		
**Variant Substitution**	**Dabrafenib**	**Prediction Dabrafenib**
L505H	Unknown	S
L597R	Unknown	R
V600E+L505H	Unknown	R
V600E+L514V	Unknown	S

S—susceptible; R—resistant.

**Table 5:** Vemurafenib BRAF variants resistance prediction.

Variants with Known Classification		
Variant Substitution	Vemurafenib	Prediction Vemurafenib
V600E	S	S
V600M	S	S
V600K	S	S
V600D	S	S
V600R	S	S
L505H	R	R
G466E	R	R
G469A	R	R
V600E+L514V	R	R
L597R	S	S
V600E+L505H	R	R
		Accuracy = 100.00%
**Variants of Unknown Significance**		
**Variant Substitution**	**Vemurafenib**	**Prediction Vemurafenib**
S467V	Unknown	S
G469V	Unknown	R
K601E	Unknown	R
L597S	Unknown	S
WT	Unknown	S

S—susceptible; R—resistant.

**Table 6:** Dabrafenib BRAF variants binding affinities.

Variants with Known Classification		
Variant Substitution	Dabrafenib	Experimental IC50
V600E	S	0.65 nm [15]
V600M	S	-
V600K	S	0.5 nm [15]
V600D	S	1.84 nm [15]
V600R	S	-
G466E	R	-
WT	S	3.2 nm [15]
L597S	S	>1 µM [16]
K601E	R	-
G469A	R	>1 µM [16]
G469V	R	-
S467L	R	-

**Table 7:** BRAF variants pathogenicity prediction for variants with known classification.

Variant Substitution	AlphaMissense	PredictSNP
G466E	likely_pathogenic	Deleterious
S467L	likely_pathogenic	Deleterious
S467V	likely_pathogenic	Deleterious
G469A	likely_pathogenic	Deleterious
G469V	likely_pathogenic	Deleterious
L505H	likely_pathogenic	Deleterious
L597R	likely_pathogenic	Deleterious
L597S	likely_pathogenic	Deleterious
V600D	likely_pathogenic	Deleterious
V600E	likely_pathogenic	Deleterious
V600K	likely_pathogenic	Deleterious
V600M	likely_pathogenic	Neutral
V600R	likely_pathogenic	Deleterious
K601E	likely_pathogenic	Deleterious
V600E+L514V	-	-
V600E+L505H	-	-
WT	-	-

The PredictSNP consensus score uses scores from MAPP, PhD-SNP, Polyphen-1, Polyphen-2, SIFT, and SNAP all of which display additional neutral predictions.

**Table 8:** Proposed list of variants of BRAF.

BRAF variants	Dabrafenib	Vemurafenib
V600E	S	S
V600M	S	S
V600K	S	S
V600D	S	S
V600R	S	S
G466E	R	R
WT	S	Unknown
L597S	S	Unknown
K601E	R	Unknown
G469A	R	R
G469V	R	Unknown
S467L	R	Unknown
L505H	Unknown	R
L597R	Unknown	S
V600E+L505H	Unknown	R
V600E+L514V	Unknown	R

S—susceptible; R—resistant.

## Data Availability

The original contributions presented in the study are included in the article, further inquiries can be directed at the corresponding author.

## Supplementary Materials

The following supporting information can be downloaded at: https://www.mdpi.com/article/10.3390/molecules30173556/s1, Figure S1. RMSD (root mean square deviation) plots for REST2 molecular dynamics simulations. The RMSD values for the trajectories have stabilized for WT and variant BRAF molecules. (A) V600E (B) V600M (C) V600K (D) V600D (E) V600R (F) G466E (G) WT (H) L597S (I) K601E (J) G469A (K) G469V (L) S467L (M) L505H (N) L597R (O) V600E+L505H (P) V600E+L514V (Q) ALL. Figure S2. Feature selection for dabrafenib using decision trees. (A) The 1st decision tree identified dihedral angles phi664 and phi600. (B) The 2nd decision tree identified dihedral angles phi663 and psi494. (C) The 3rd decision tree identified dihedral angles psi675 and phi677. Figure S3. Feature selection for vemurafenib using decision trees. (A) The 1st decision tree identified dihedral angles psi622. (B) The 2nd decision tree identified dihedral angles phi484. (C) The 3rd decision tree identified dihedral angles psi450 and phi495. (D) The 4th decision tree identified dihedral angles phi518 and phi622.
