# Supplementary material for: Combining Molecular Dynamics and Machine Learning to Predict Drug Resistance Causing Variants of BRAF in Colorectal Cancer
Source: Molecules. 2025 Aug 30;30(17):3556. doi: 10.3390/molecules30173556 (PMC12430524; doi:10.3390/molecules30173556)
Supplement: Supplementary file 1 [file molecules-30-03556-s001.zip › Supplemental Figures.pdf]

Supplemental Material

(A) V600E

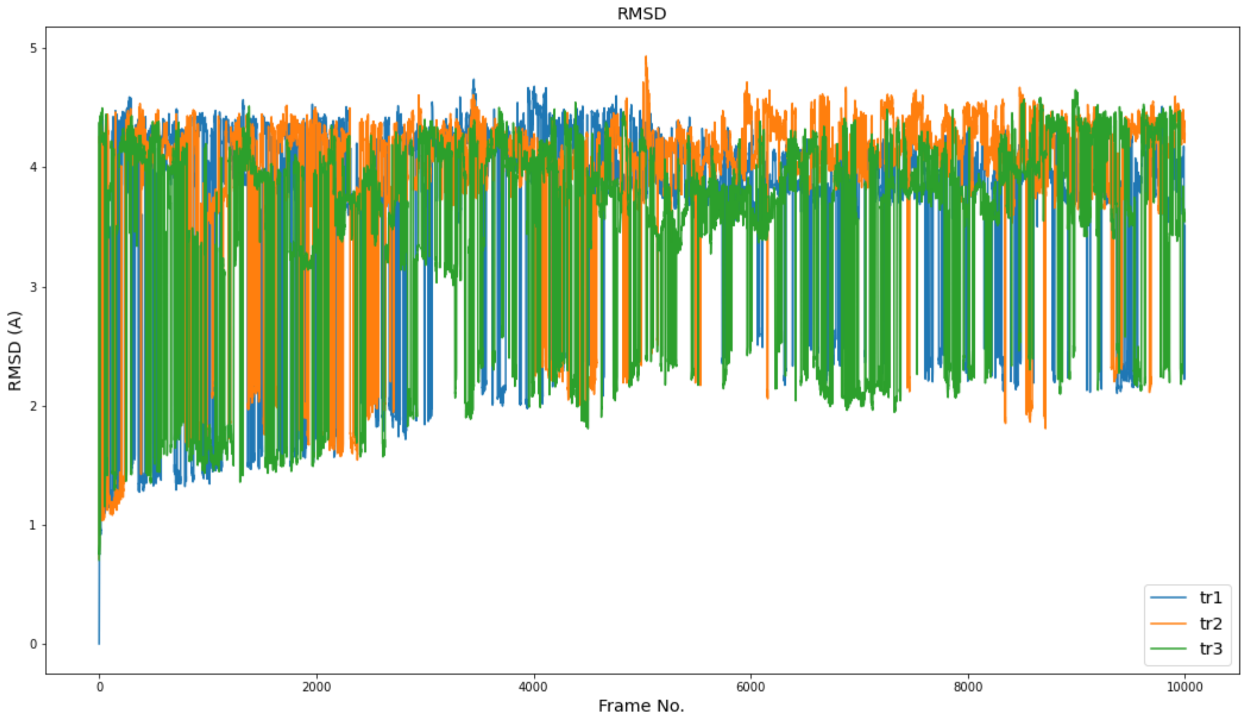

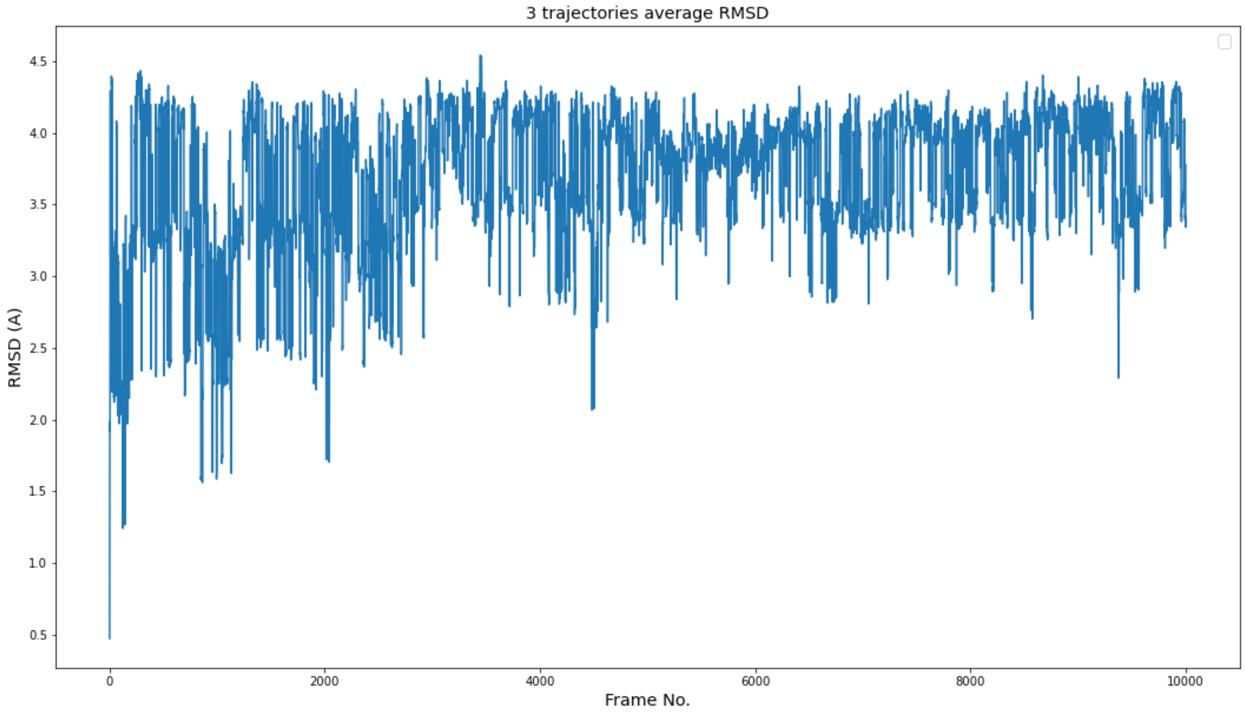

(B) V600M

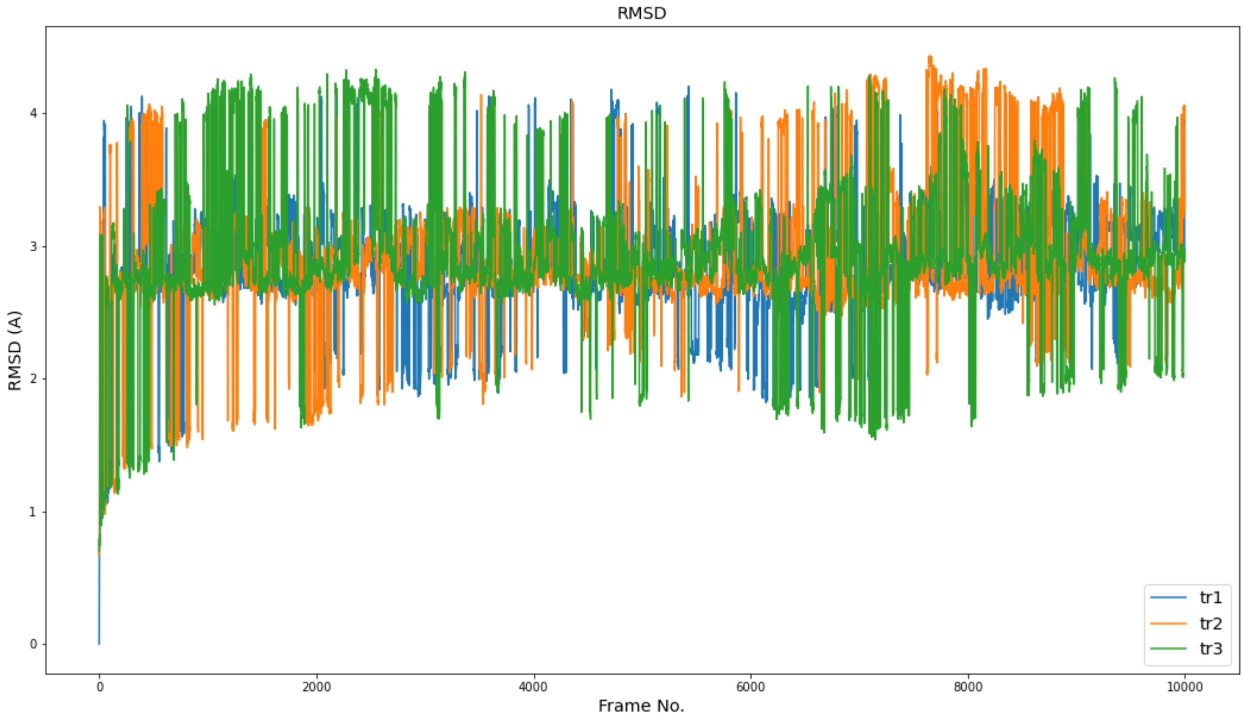

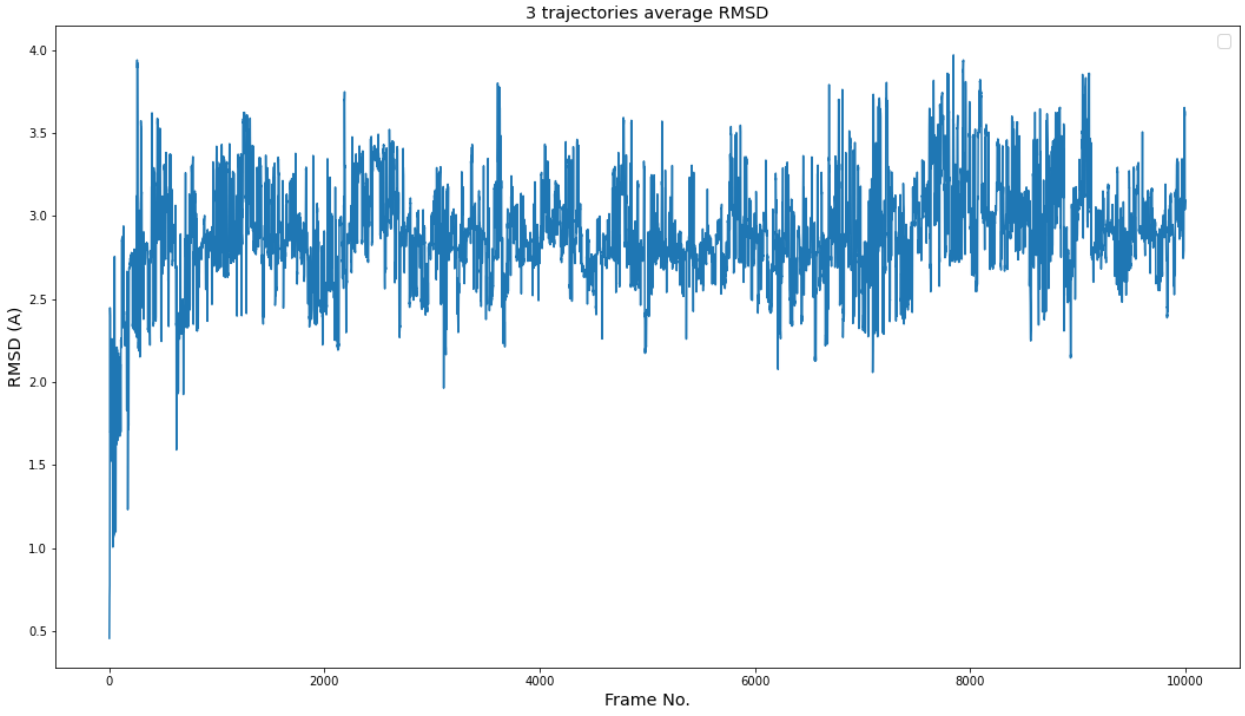

(C) V600K

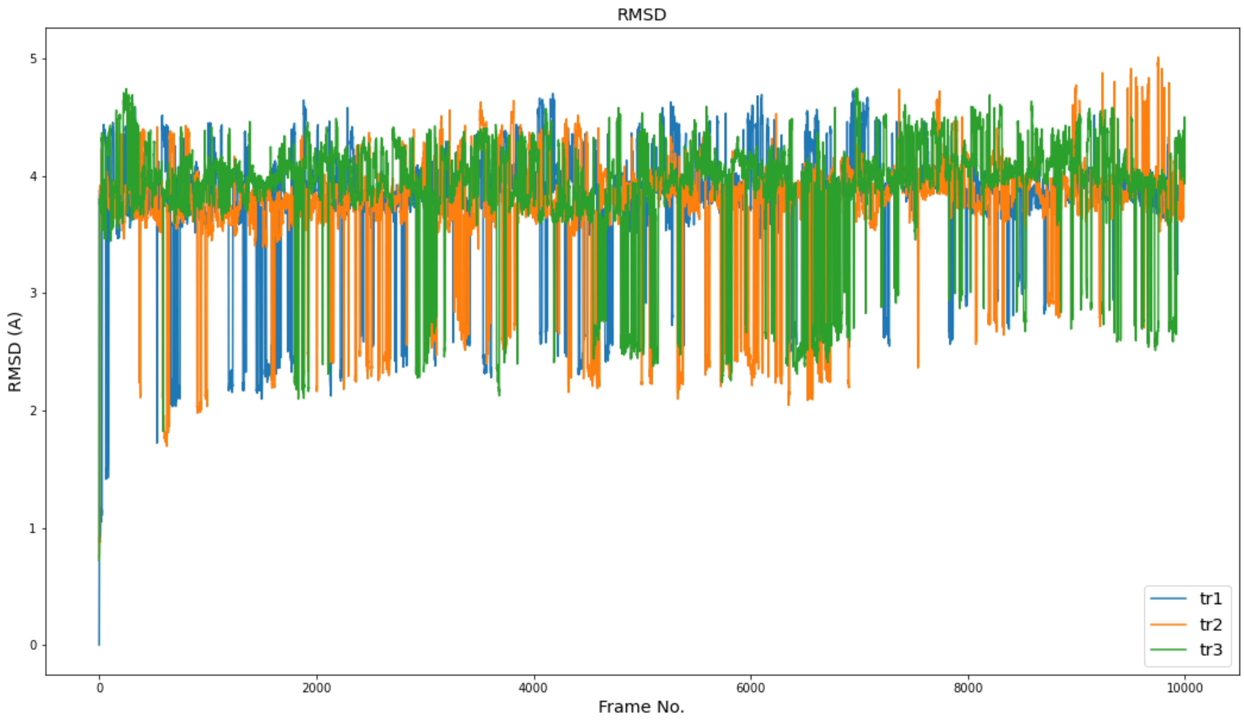

RMSD
RMSD (A)
Frame No.
tr1
tr2
tr3

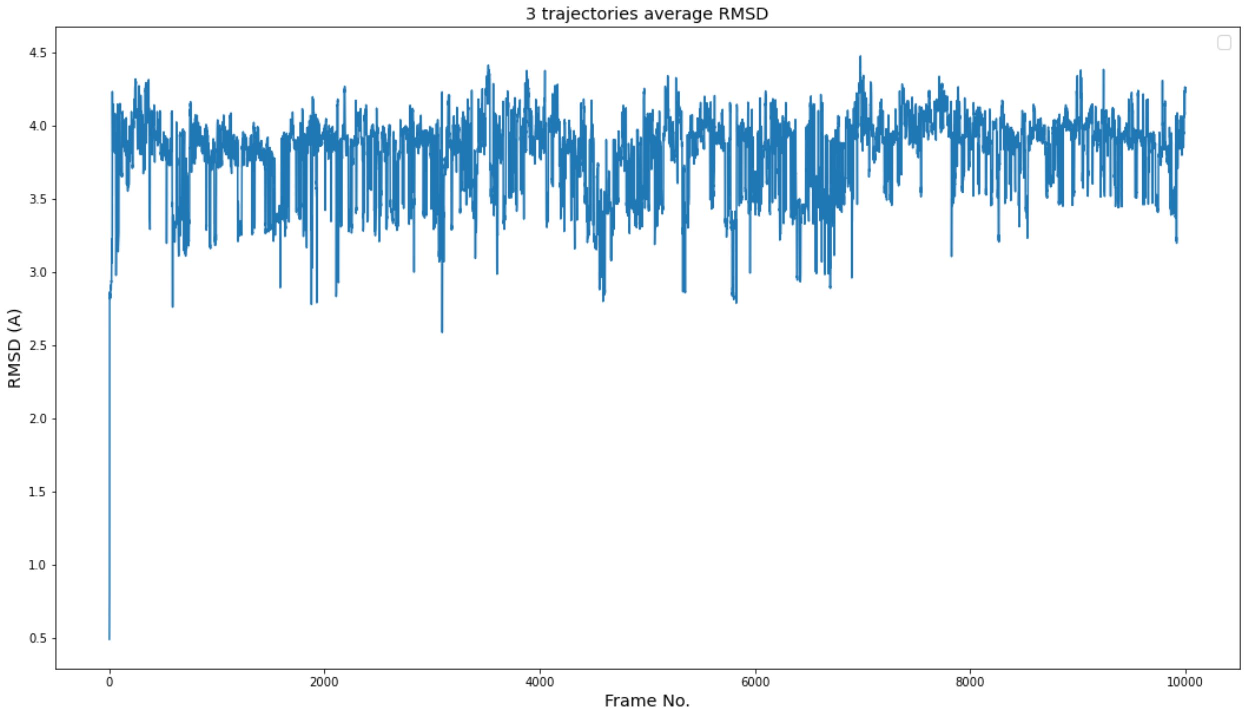

3 trajectories average RMSD
RMSD (A)
Frame No.

(D) V600D

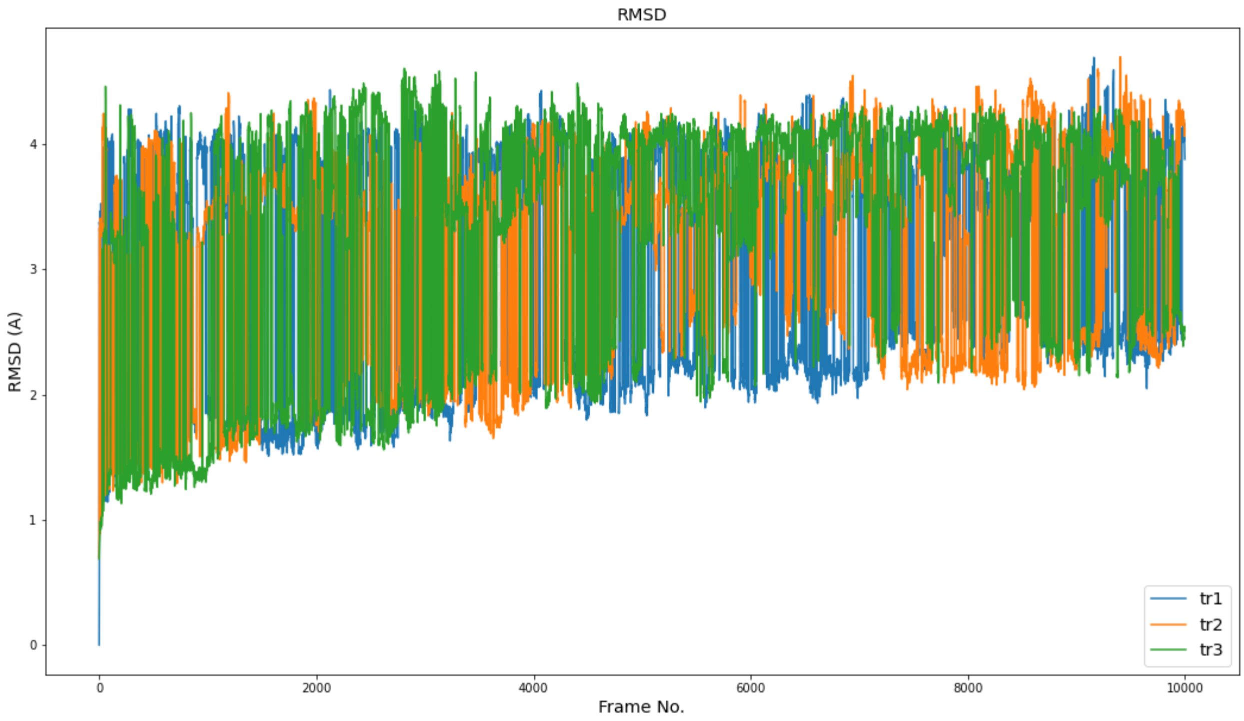
RMSD
RMSD (A)
Frame No.
tr1
tr2
tr3
0
1
2
3
4
0
2000
4000
6000
8000
10000

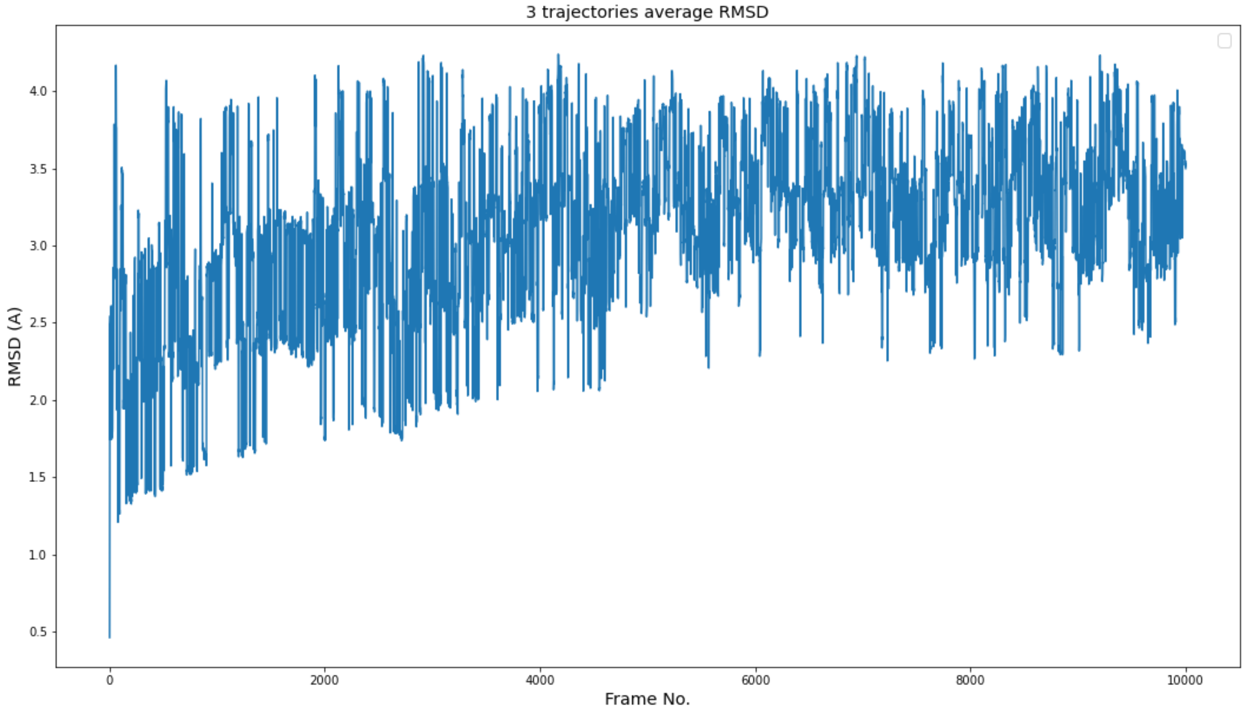
3 trajectories average RMSD
RMSD (A)
Frame No.
0.5
1.0
1.5
2.0
2.5
3.0
3.5
4.0
0
2000
4000
6000
8000
10000

(E) V600R

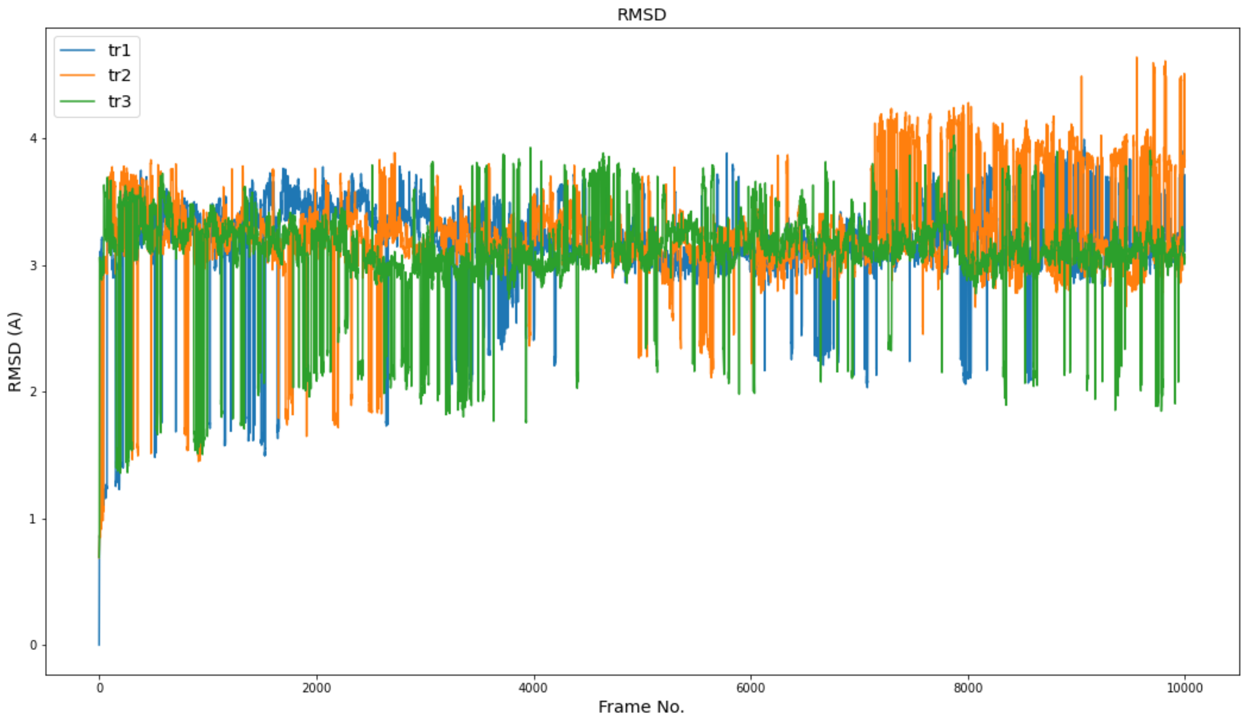
RMSD
tr1
tr2
tr3
RMSD (A)
Frame No.
0
1
2
3
4
0
2000
4000
6000
8000
10000

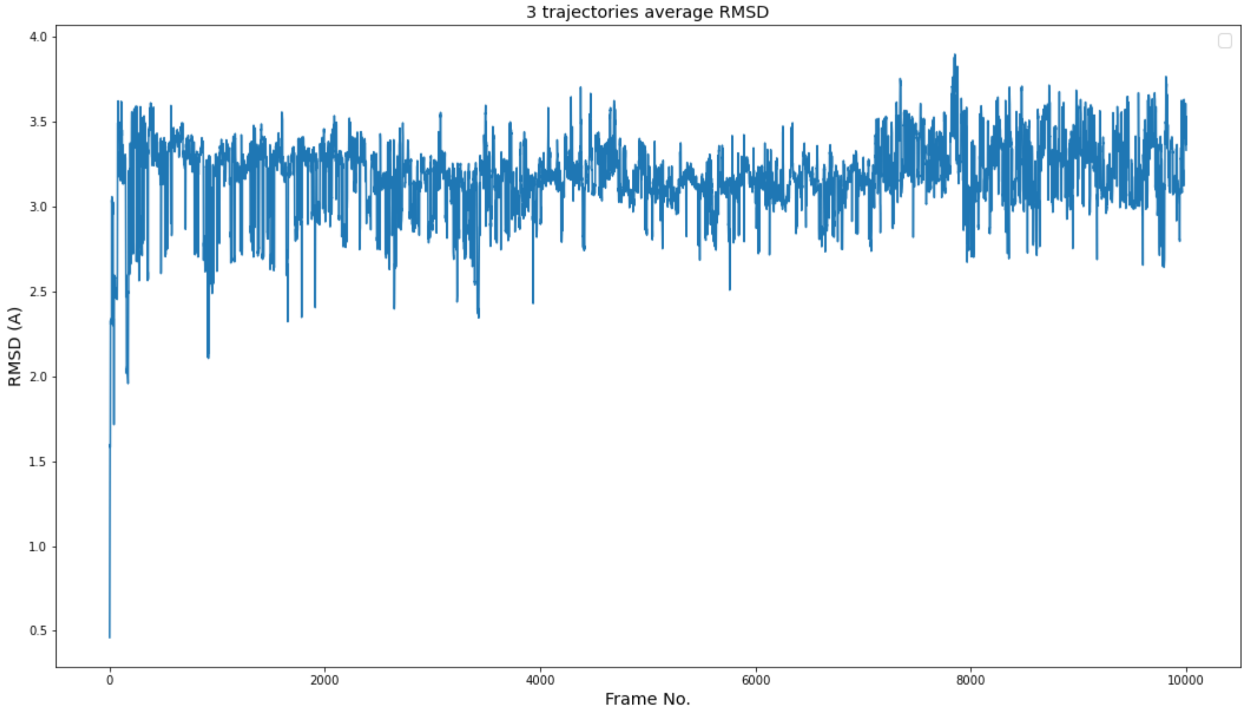
3 trajectories average RMSD
RMSD (A)
Frame No.
0.5
1.0
1.5
2.0
2.5
3.0
3.5
4.0
0
2000
4000
6000
8000
10000

(F) G466E

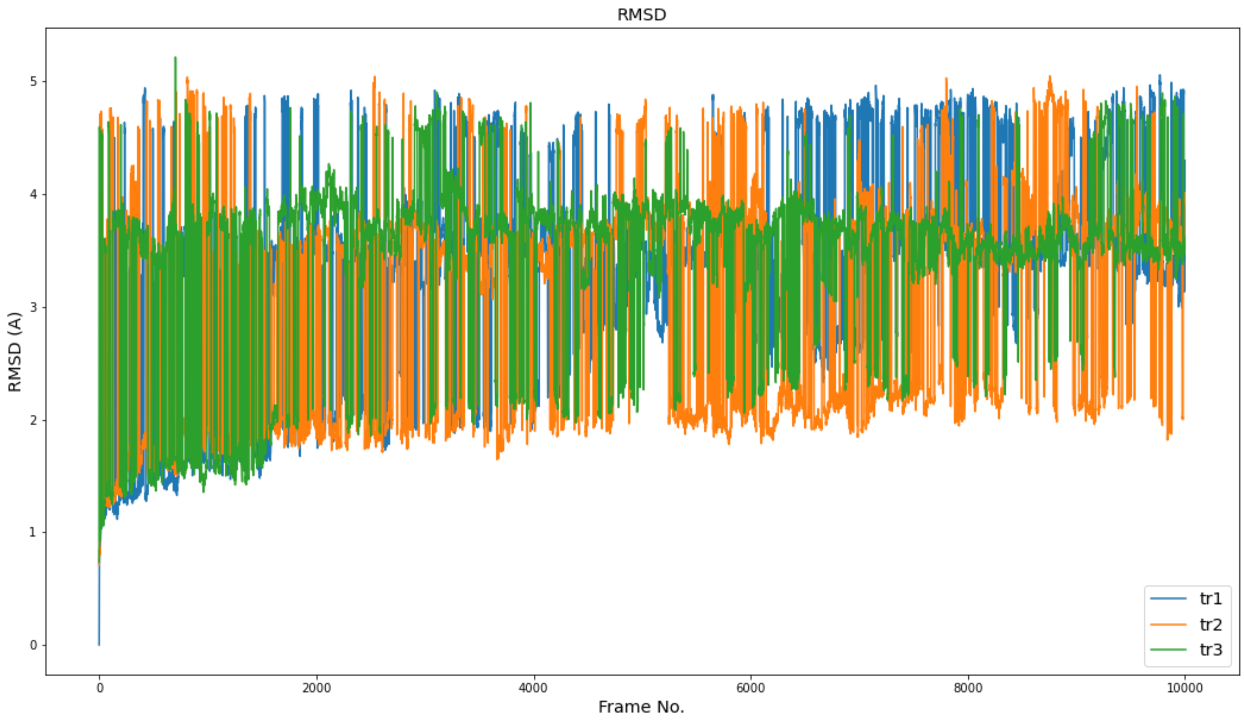
RMSD
RMSD (A)
Frame No.
tr1
tr2
tr3
0
1
2
3
4
5
0
2000
4000
6000
8000
10000

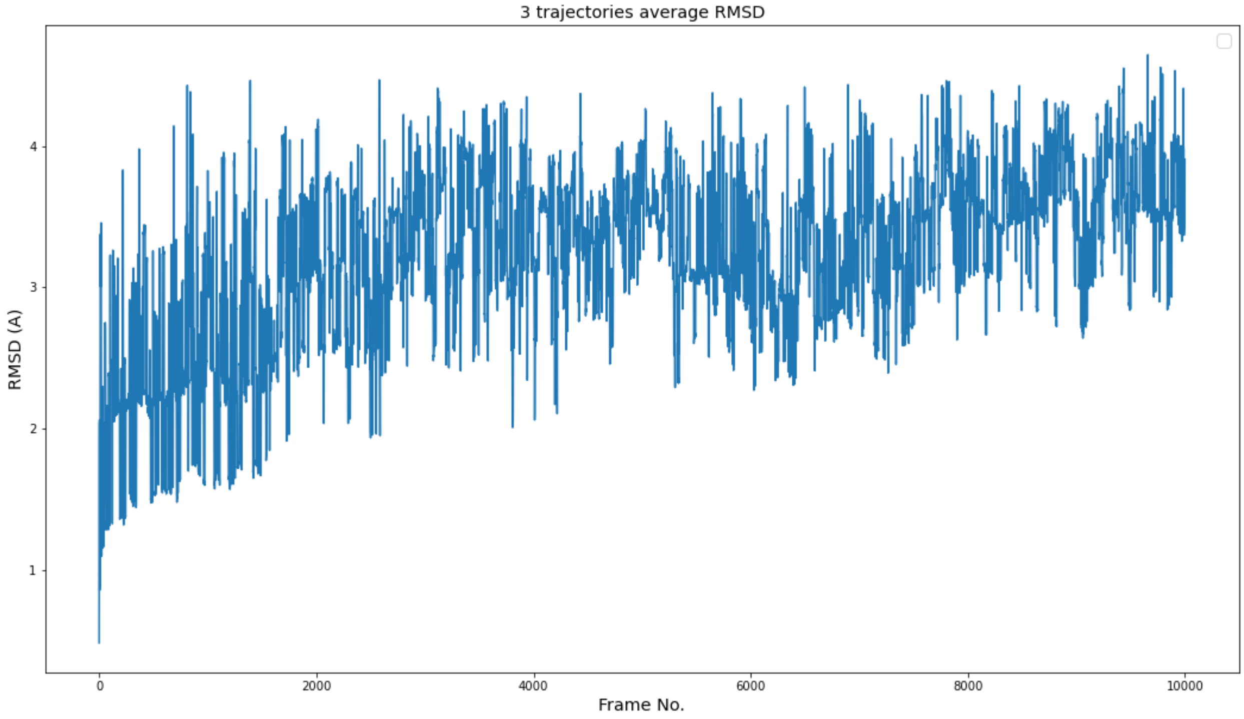
3 trajectories average RMSD
RMSD (A)
Frame No.
1
2
3
4
0
2000
4000
6000
8000
10000

(G) WT

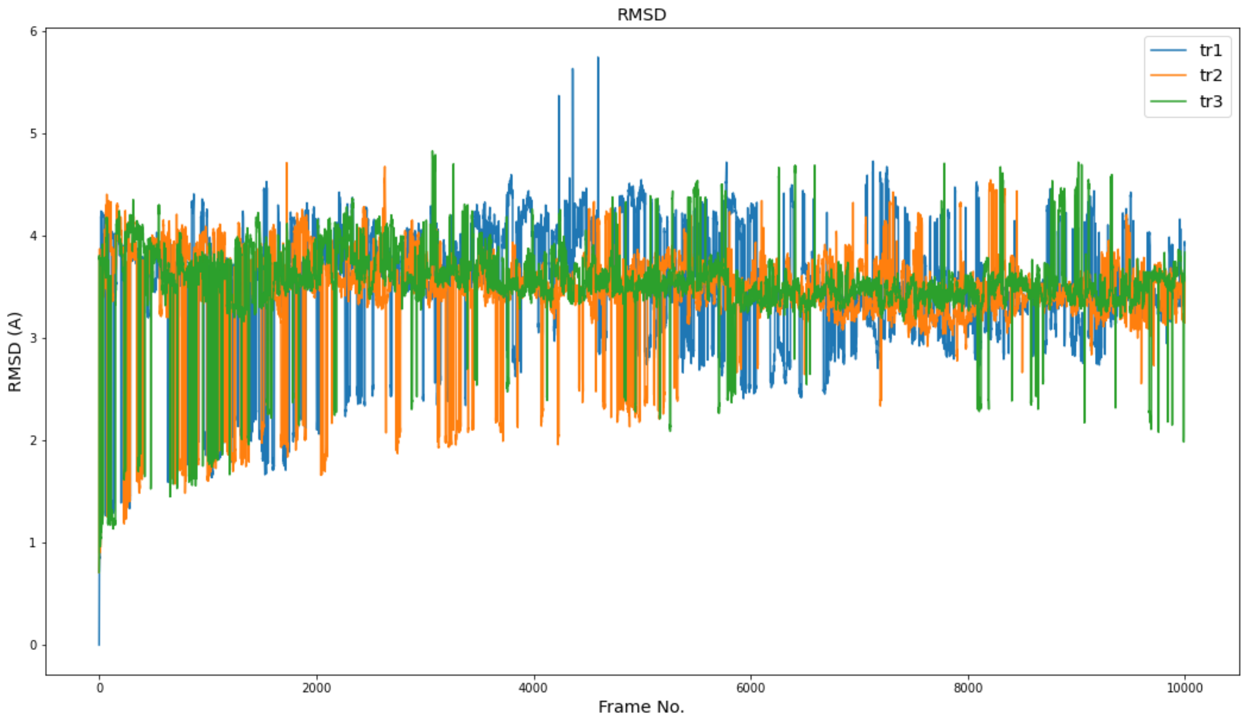
RMSD
RMSD (A)
Frame No.
tr1
tr2
tr3
0
1
2
3
4
5
6
0
2000
4000
6000
8000
10000

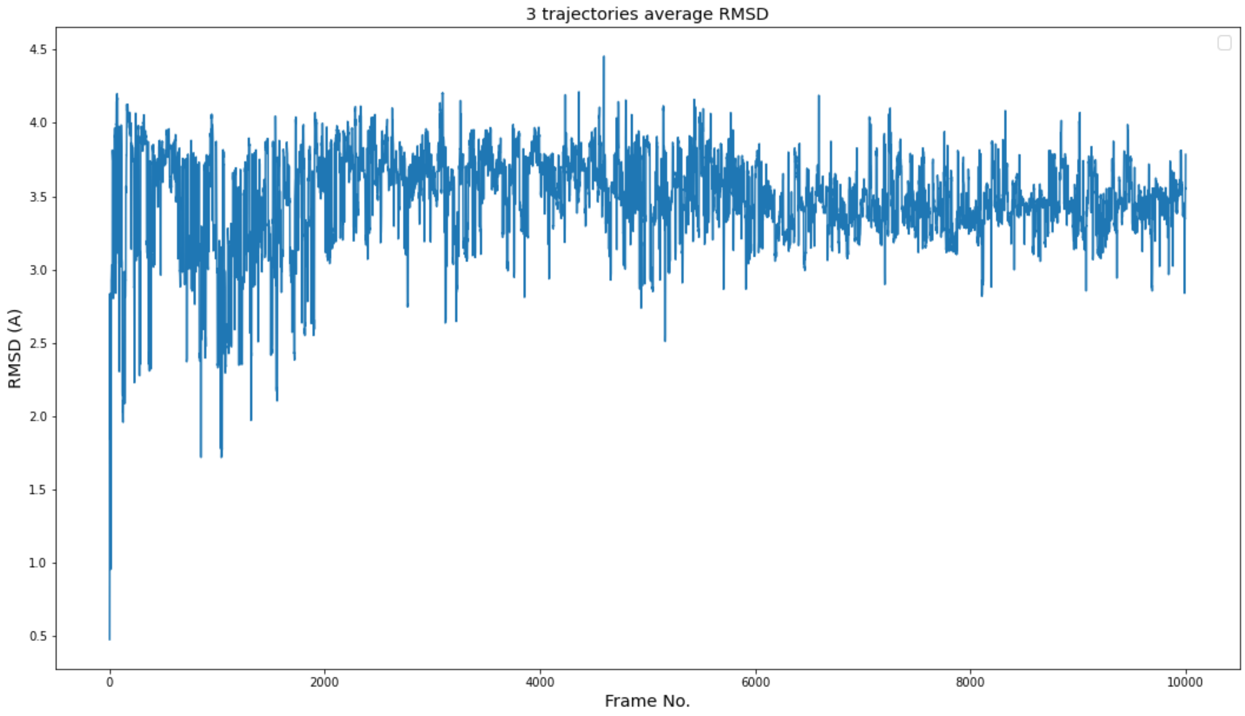
3 trajectories average RMSD
RMSD (A)
Frame No.
0.5
1.0
1.5
2.0
2.5
3.0
3.5
4.0
4.5
0
2000
4000
6000
8000
10000

(H) L597S

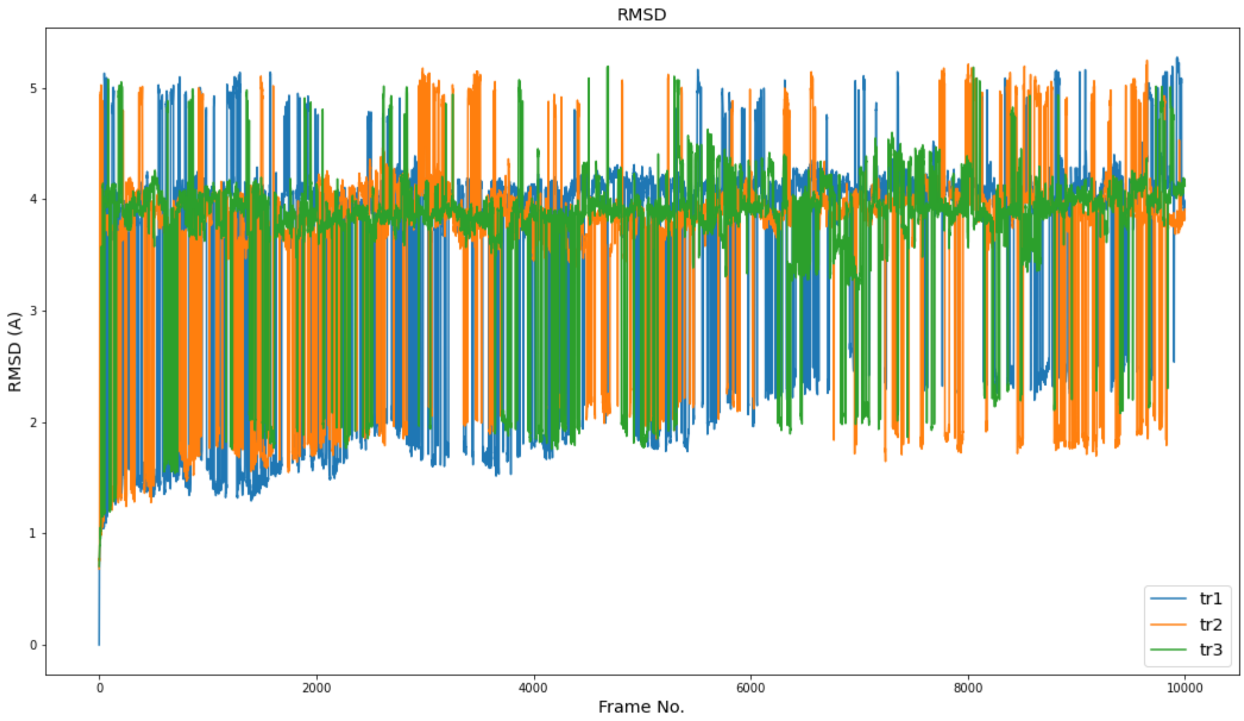
RMSD
RMSD (A)
Frame No.
tr1
tr2
tr3
0
1
2
3
4
5
0
2000
4000
6000
8000
10000

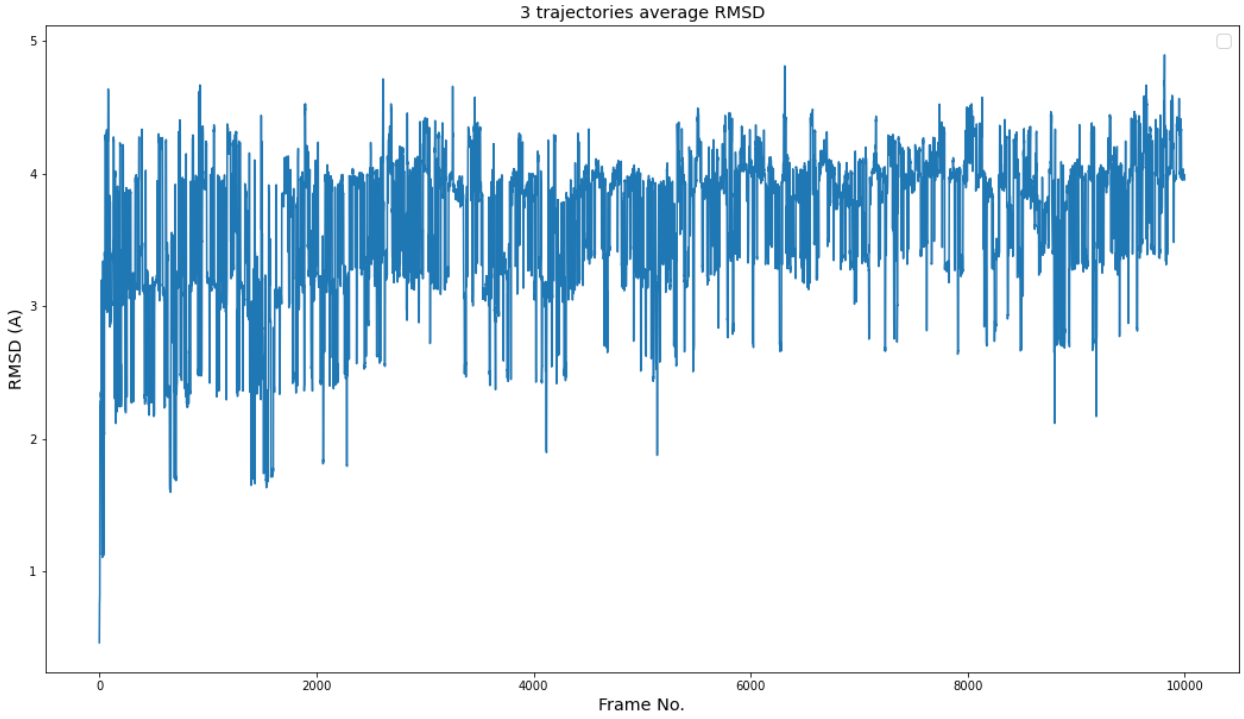
3 trajectories average RMSD
RMSD (A)
Frame No.
1
2
3
4
5
0
2000
4000
6000
8000
10000

(I) K601E

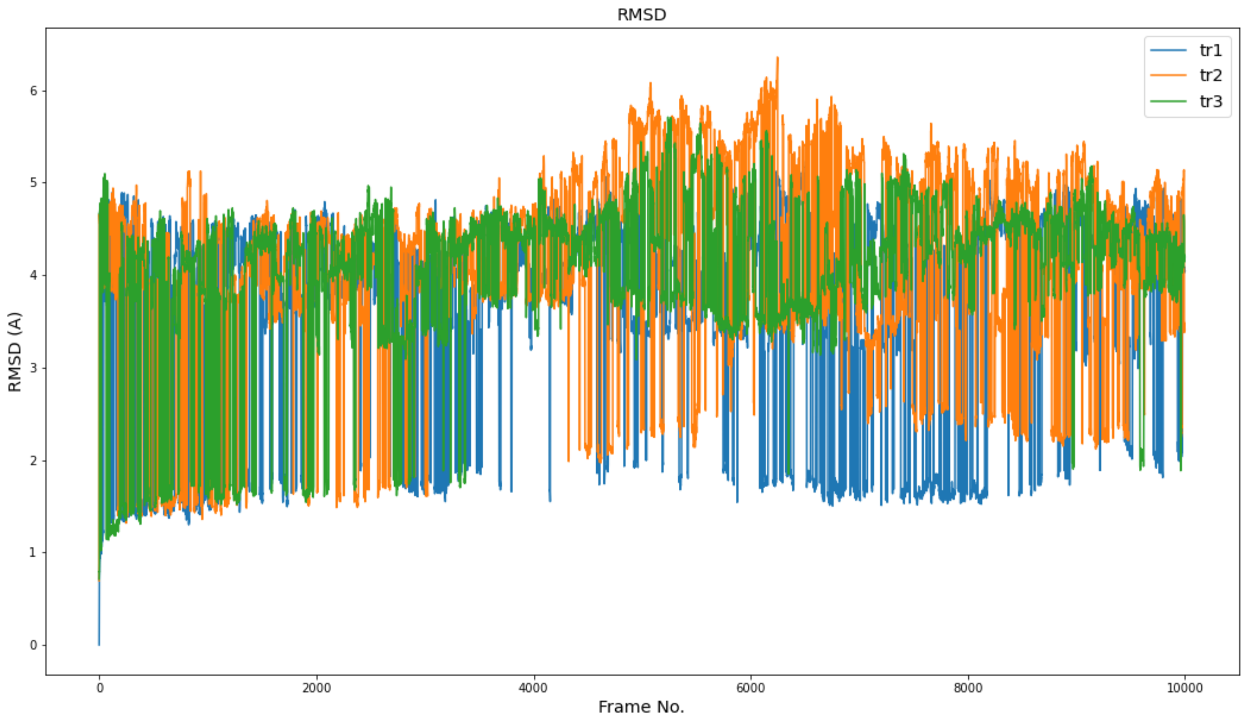
RMSD
tr1
tr2
tr3
RMSD (A)
Frame No.

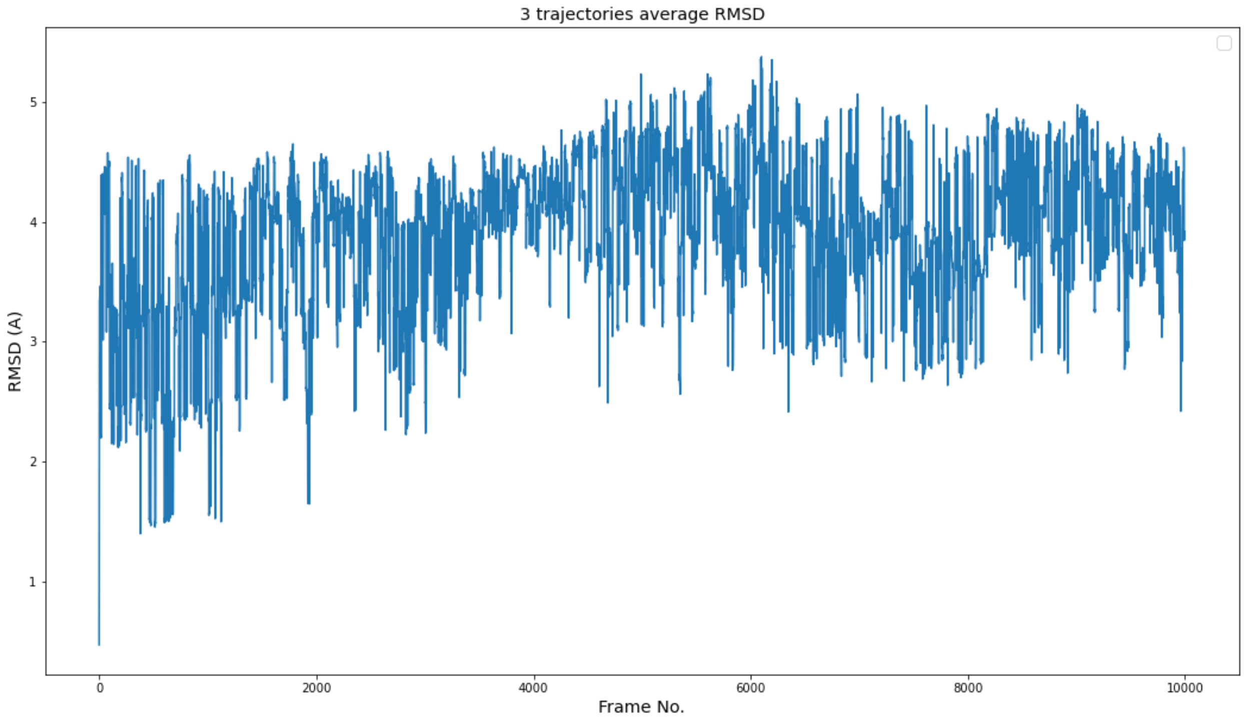
3 trajectories average RMSD
RMSD (A)
Frame No.

(J) G469A

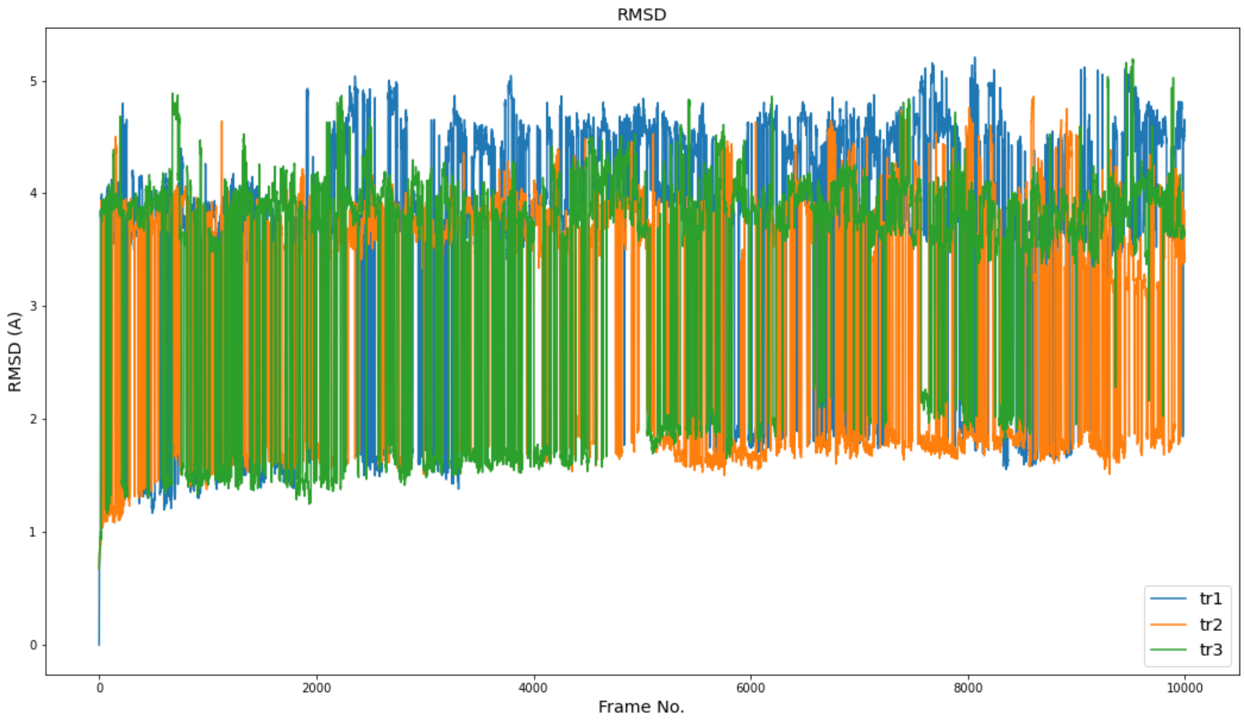
RMSD
RMSD (A)
Frame No.
0
1
2
3
4
5
0
2000
4000
6000
8000
10000
tr1
tr2
tr3

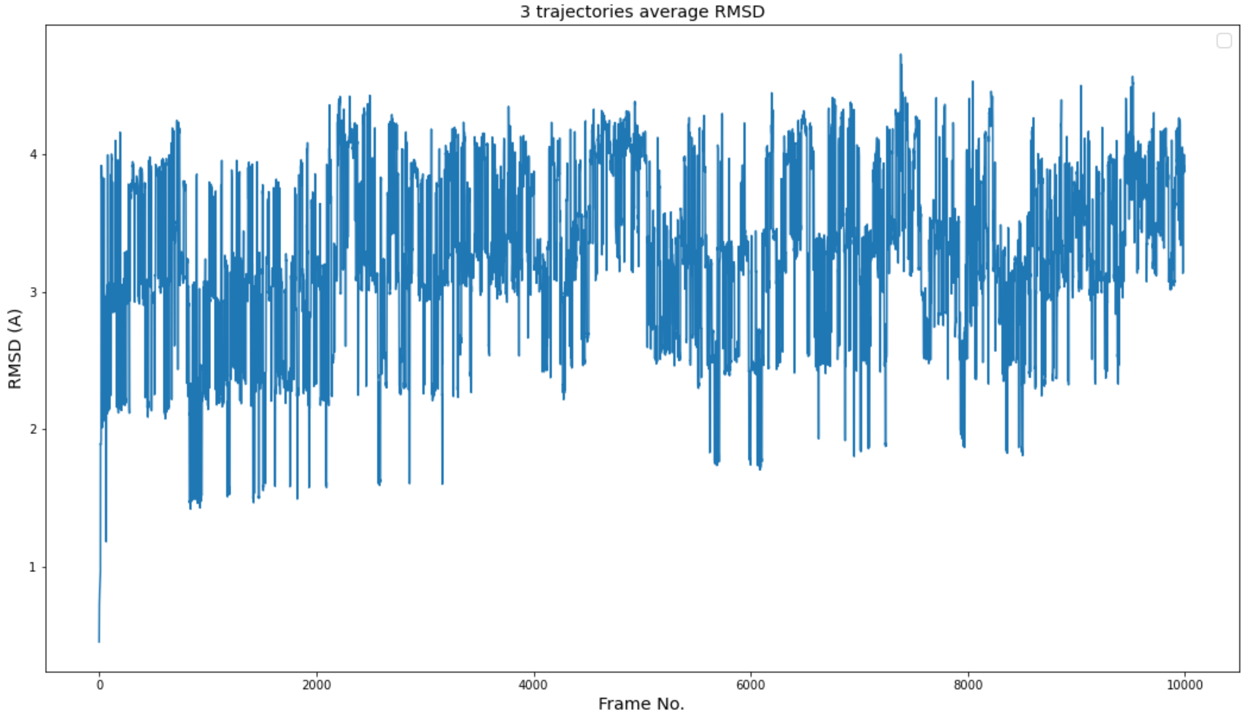
3 trajectories average RMSD
RMSD (A)
Frame No.
1
2
3
4
0
2000
4000
6000
8000
10000

(K) G469V

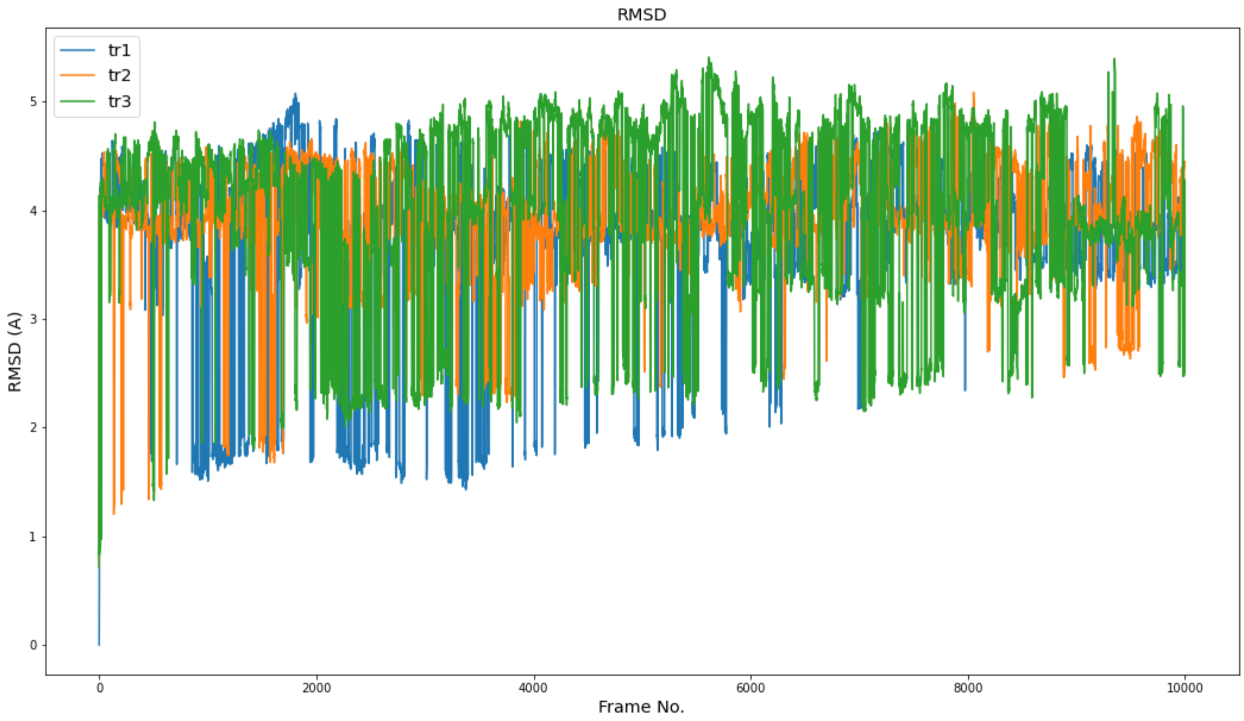
RMSD
tr1
tr2
tr3
RMSD (A)
Frame No.

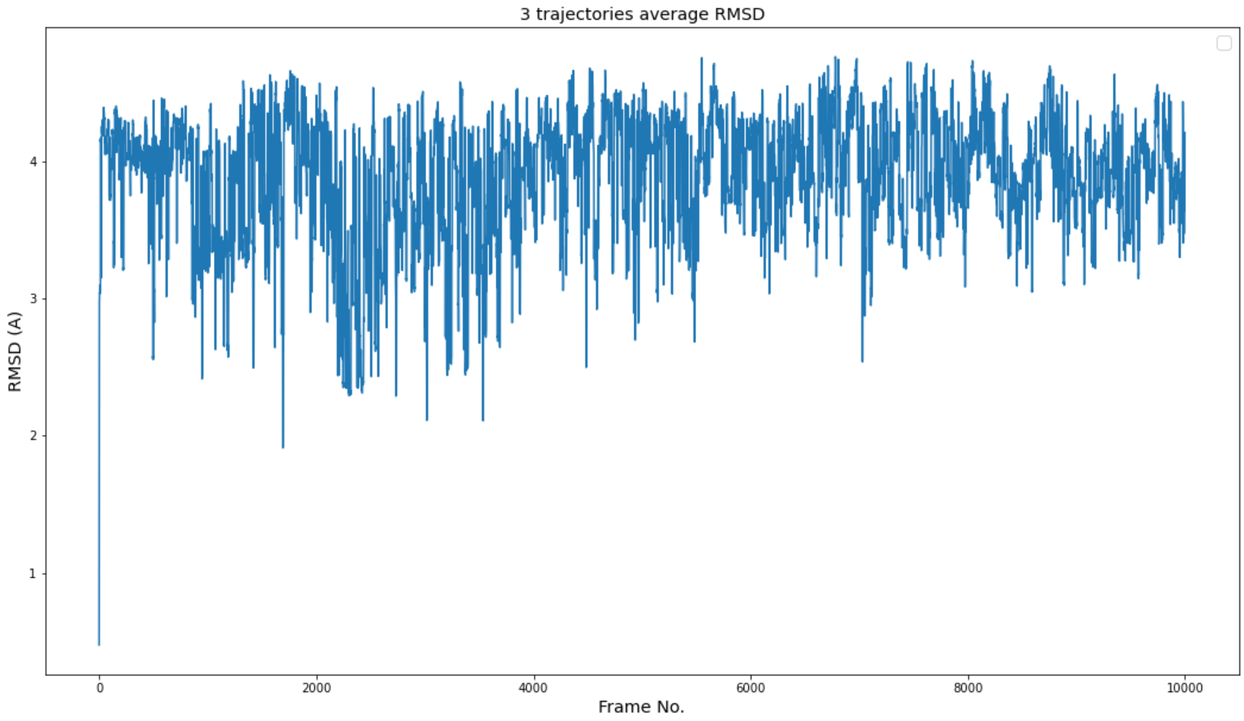
3 trajectories average RMSD
RMSD (A)
Frame No.

(L) S467L

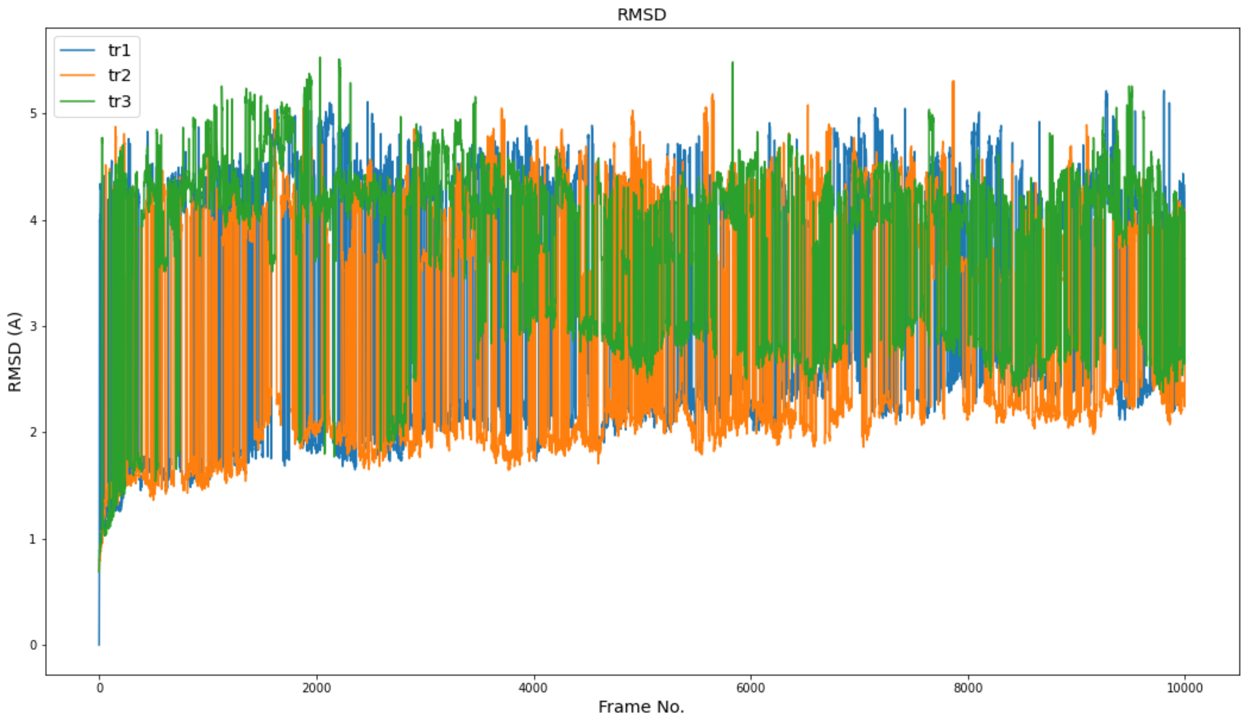
RMSD
tr1
tr2
tr3
RMSD (A)
Frame No.
0
1
2
3
4
5
0
2000
4000
6000
8000
10000

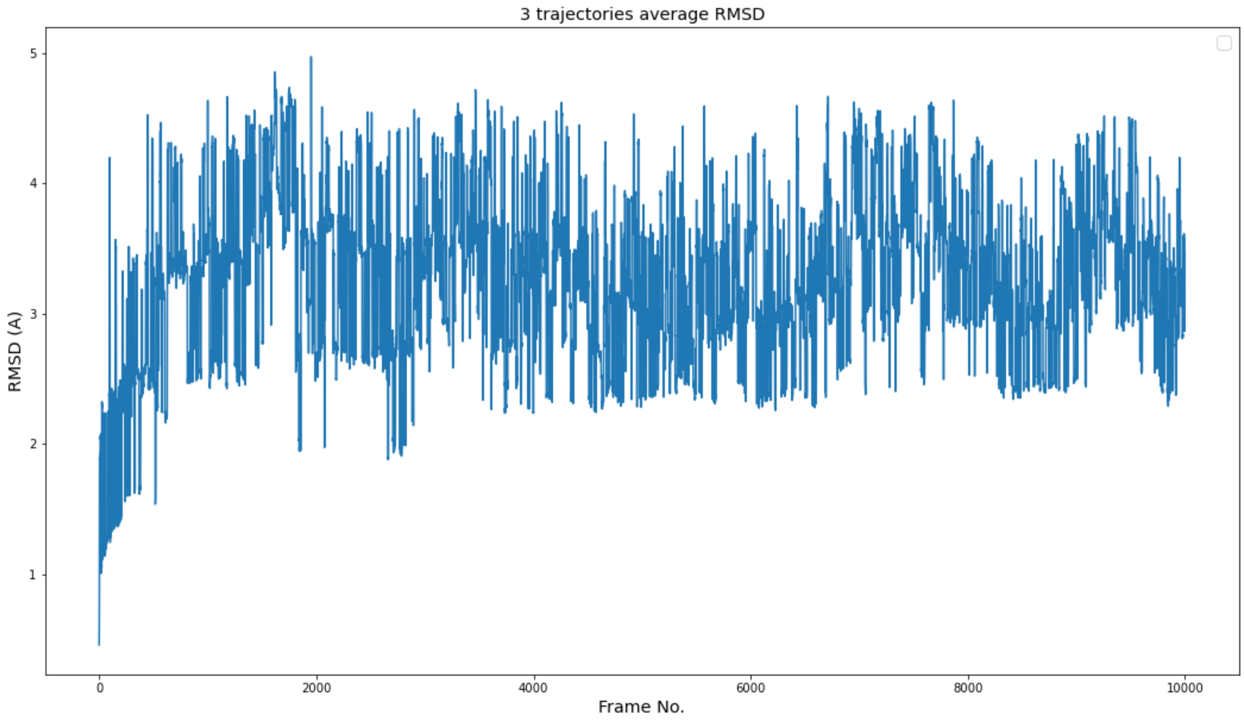
3 trajectories average RMSD
RMSD (A)
Frame No.
1
2
3
4
5
0
2000
4000
6000
8000
10000

(M) L505H

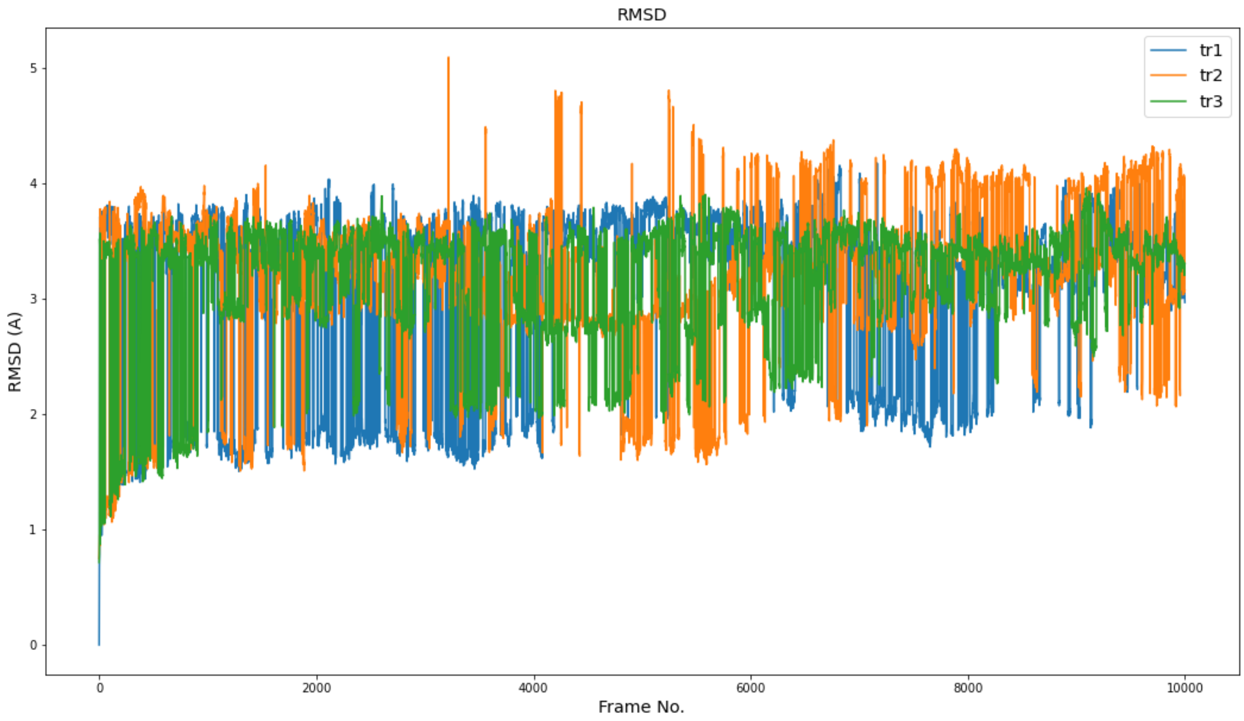
RMSD
tr1
tr2
tr3
RMSD (A)
Frame No.
0
1
2
3
4
5
0
2000
4000
6000
8000
10000

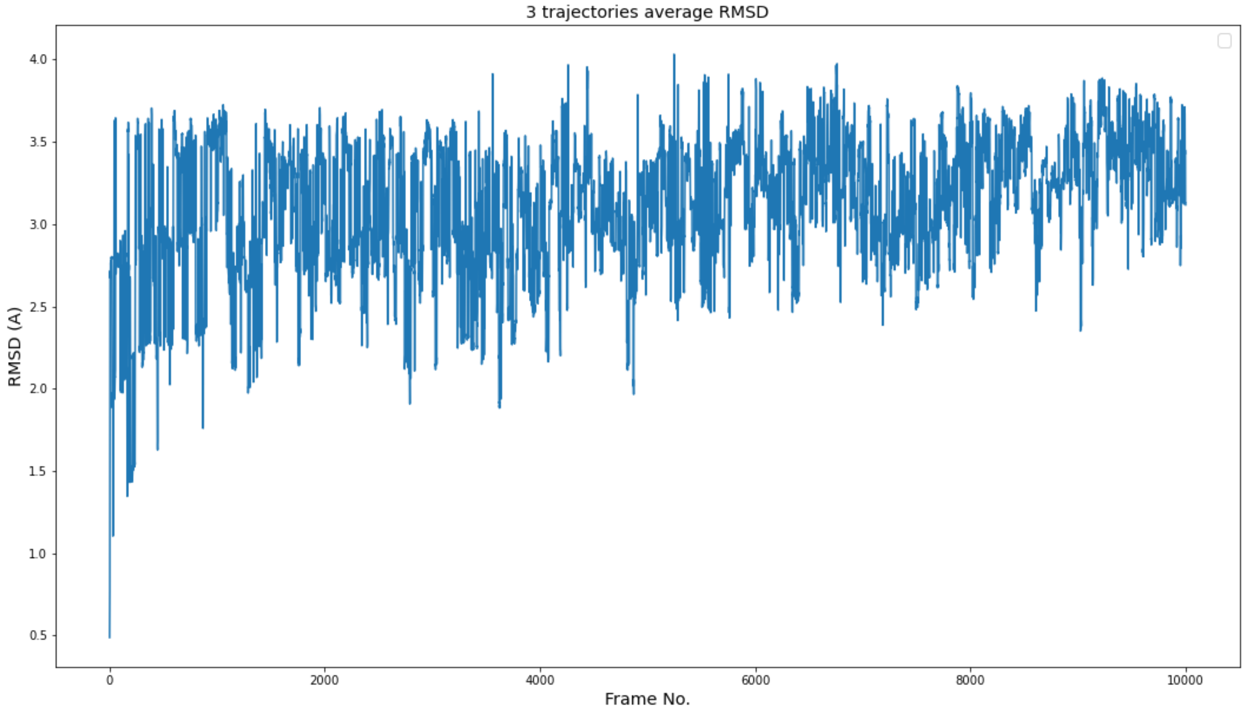
3 trajectories average RMSD
RMSD (A)
Frame No.
0.5
1.0
1.5
2.0
2.5
3.0
3.5
4.0
0
2000
4000
6000
8000
10000

(N) L597R

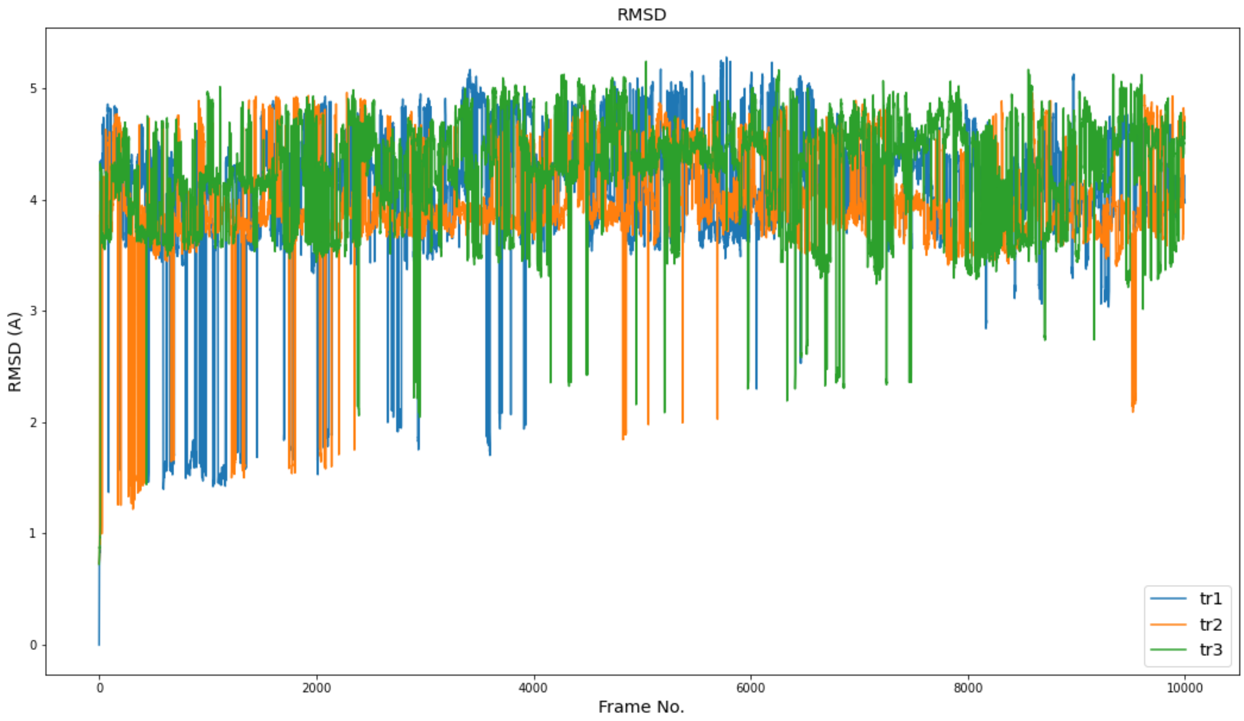

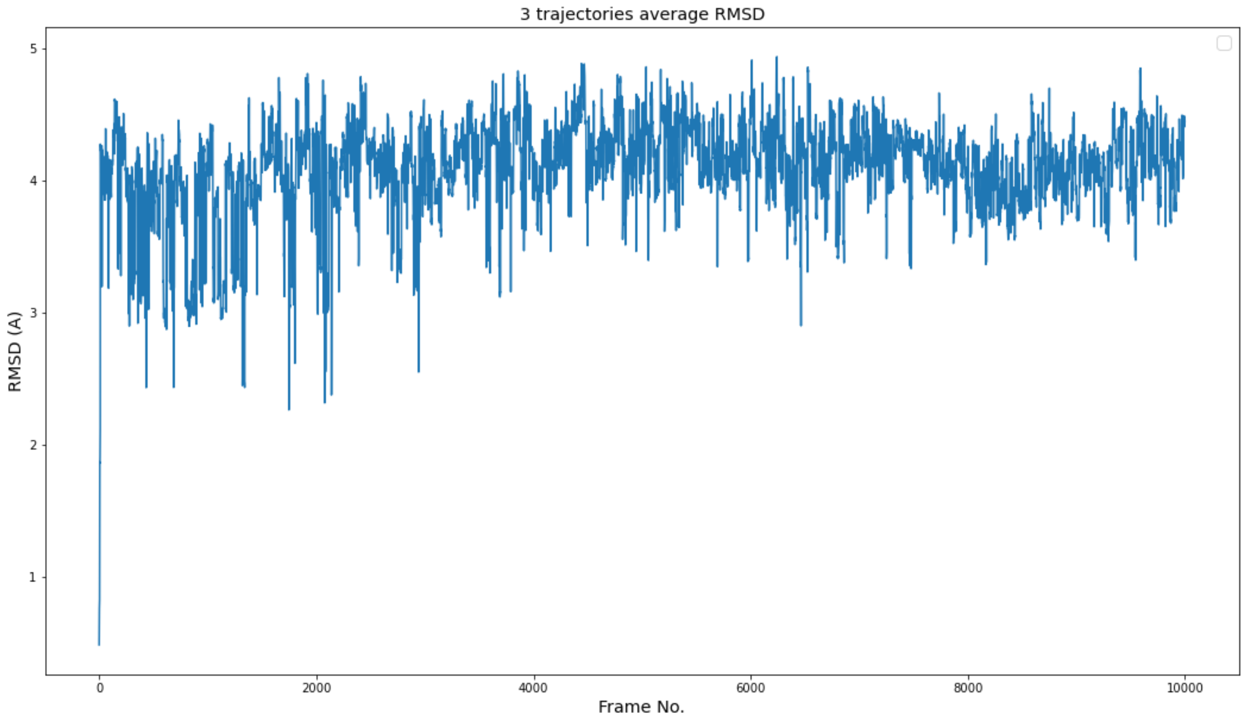

(O) V600E+L505H

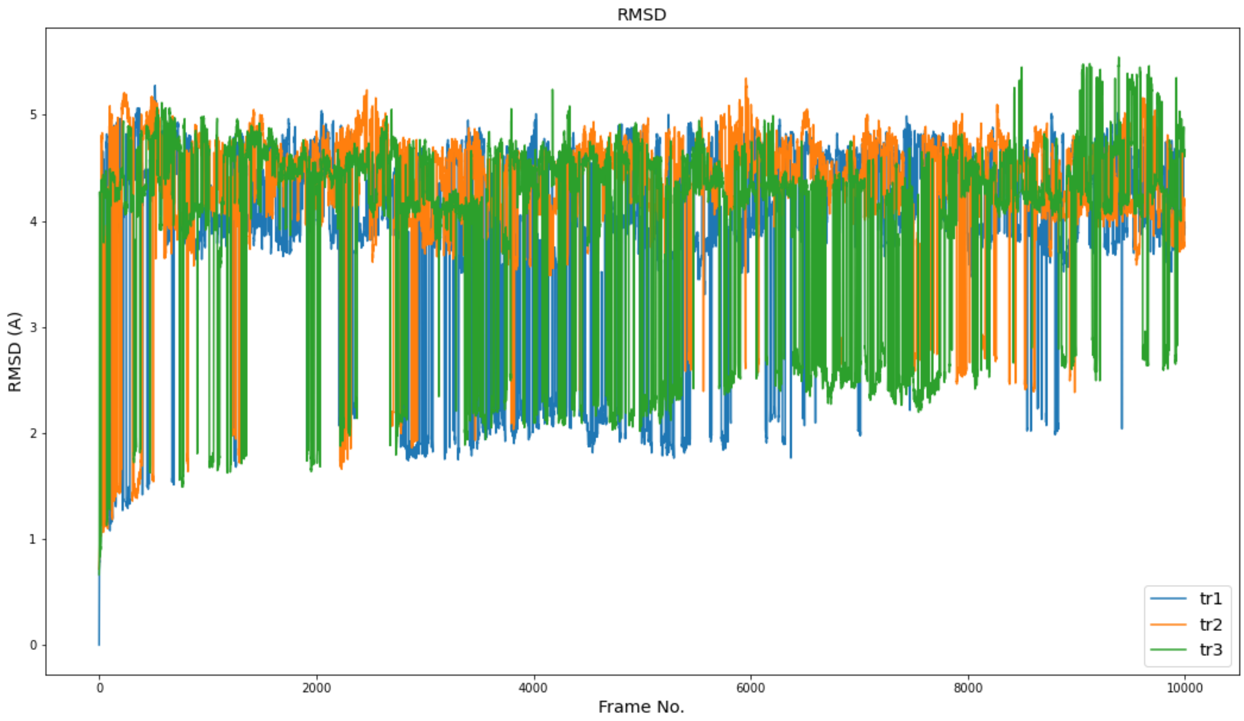

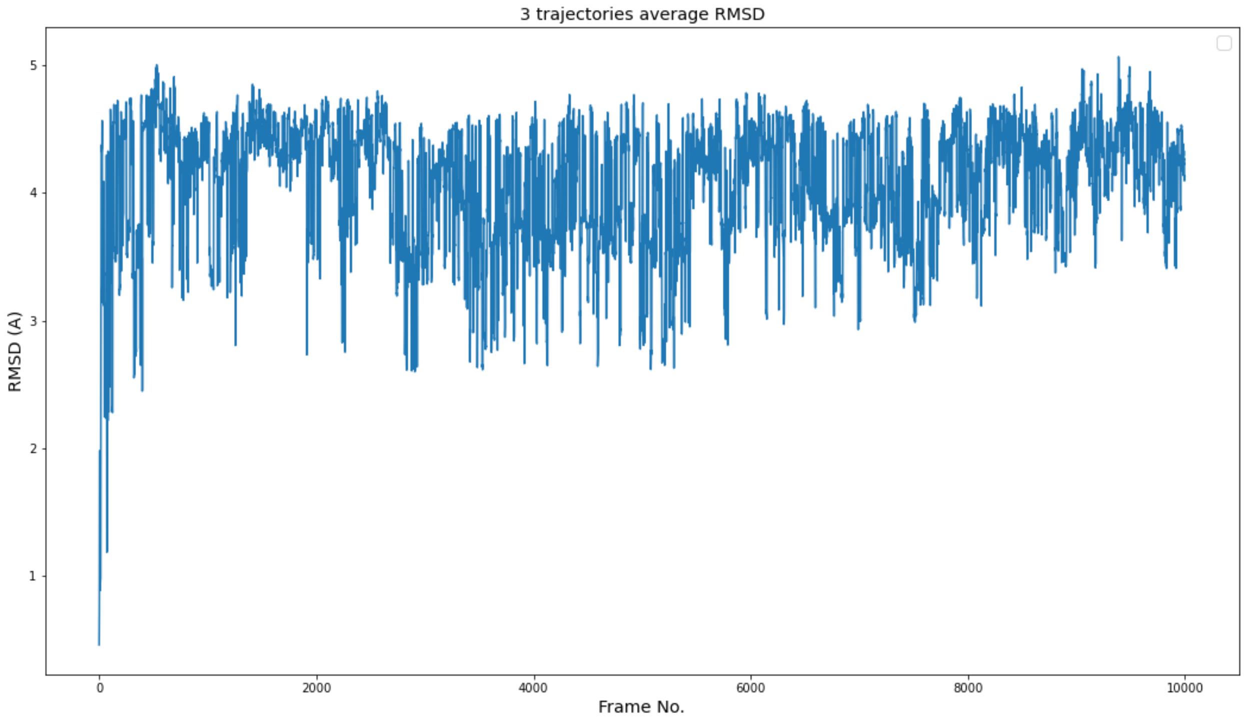

(P) V600E+L514V

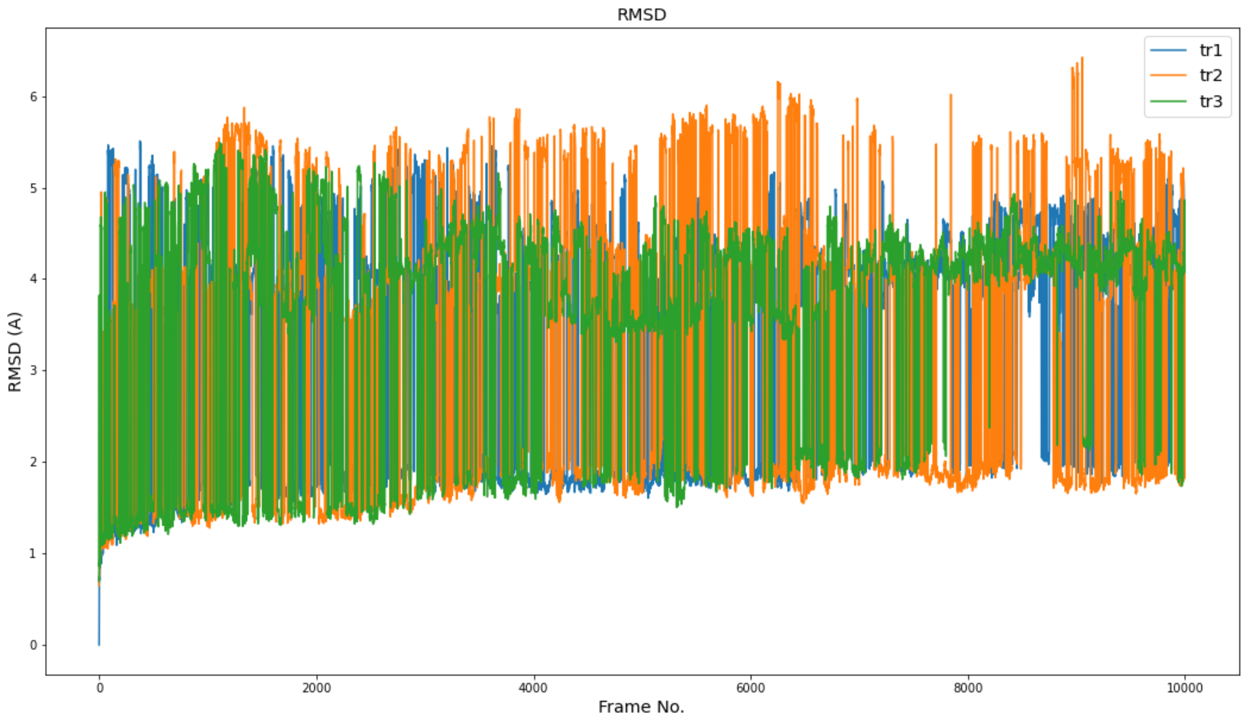
RMSD
tr1
tr2
tr3
RMSD (A)
Frame No.

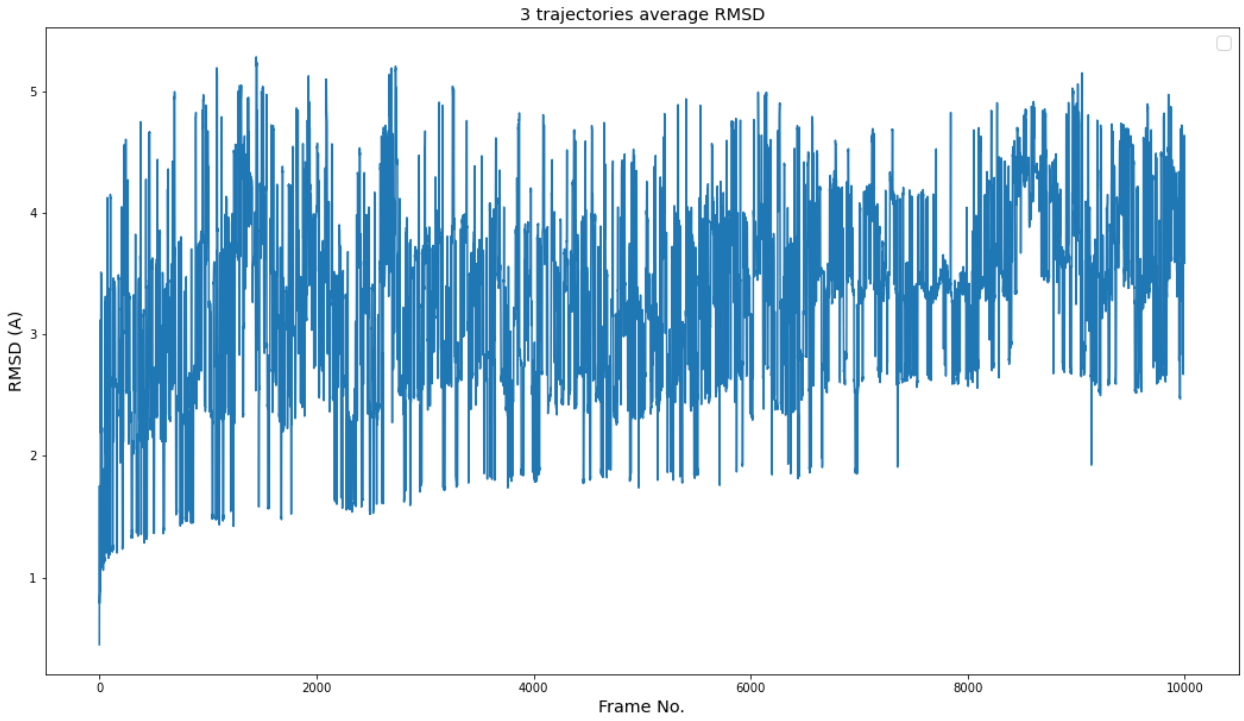
3 trajectories average RMSD
RMSD (A)
Frame No.

(Q) ALL

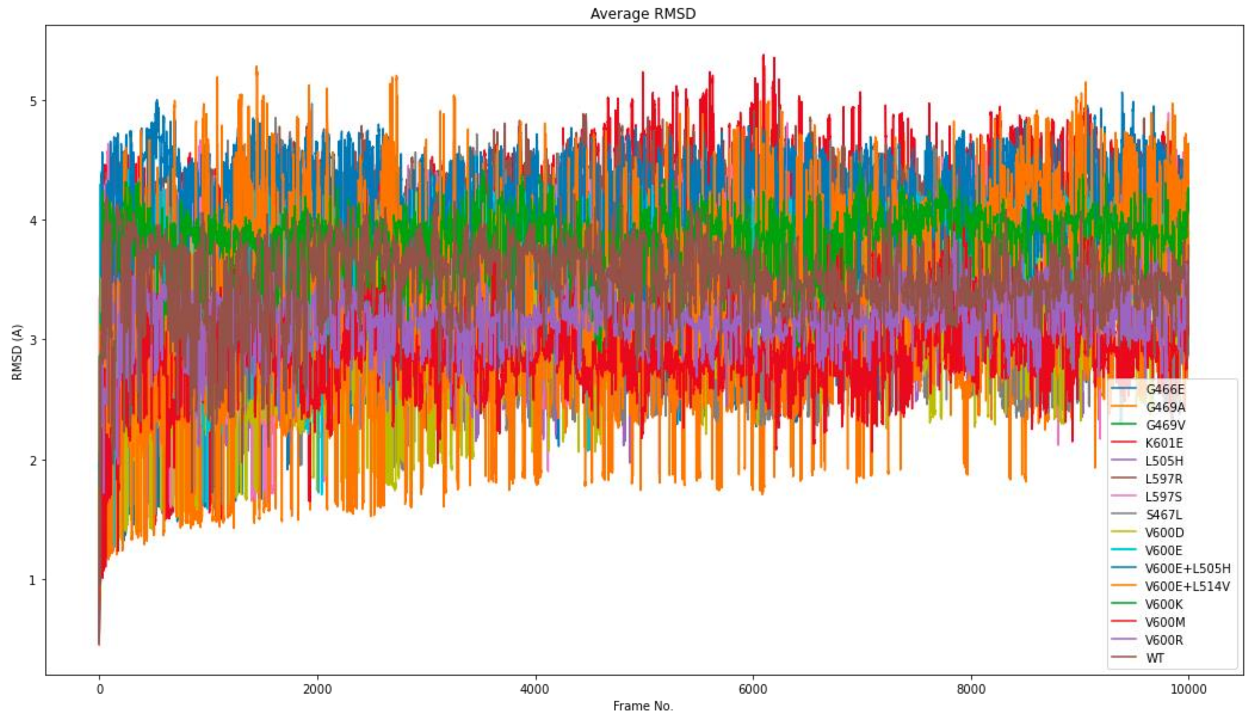

Figure S1. RMSD (root mean square deviation) plots for REST2 molecular dynamics simulations. The RMSD values for the trajectories have stabilized for WT and variant BRAF molecules. (A) V600E (B) V600M (C) V600K (D) V600D (E) V600R (F) G466E (G) WT (H) L597S (I) K601E (J) G469A (K) G469V (L) S467L (M) L505H (N) L597R (O) V600E+L505H (P) V600E+L514V (Q) ALL.

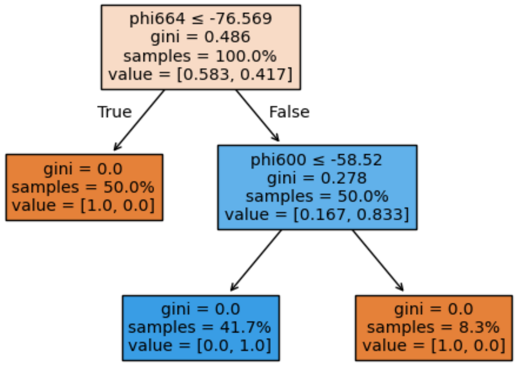

(A)

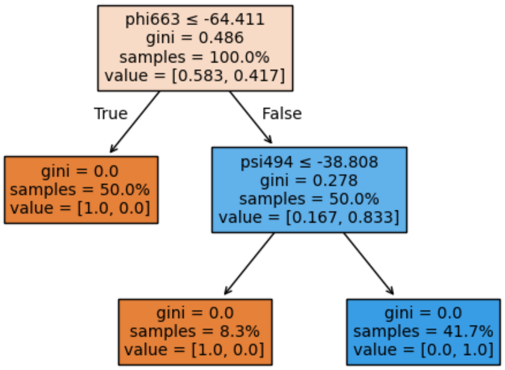

(B)

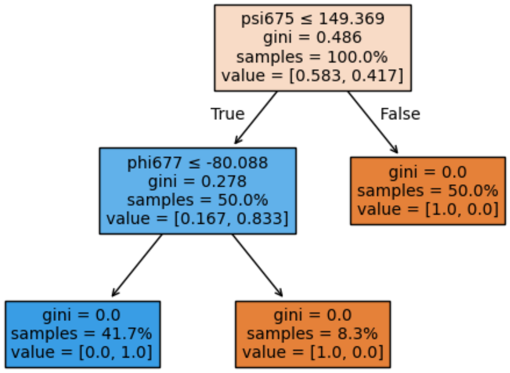

(C)

Figure S2. Decision Trees Used for Feature Selection for Dabrafenib Machine Learning Models. Feature selection for dabrafenib using decision trees. (A) The 1st decision tree identified dihedral angles phi664, and phi600. (B) The 2nd decision tree identified dihedral angles phi663 and psi494. (C) The 3rd decision tree identified dihedral angles psi675 and phi677. Each decision tree node shows the decision assessed at that node, its corresponding Gini coefficient, the percentage of samples subjected to this condition, and the repartitioning of those samples into classes. Node colors indicate the most probable class, where orange represents drug resistance for dabrafenib, blue represents drug sensitive for dabrafenib.

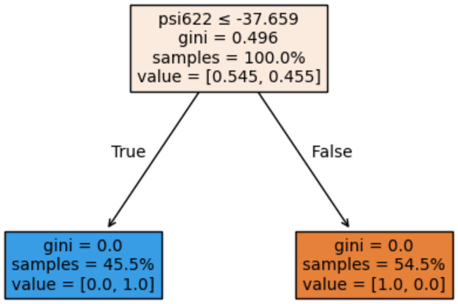

(A)

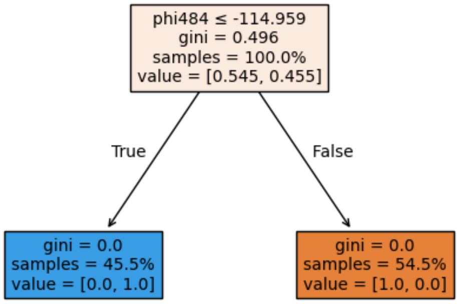

(B)

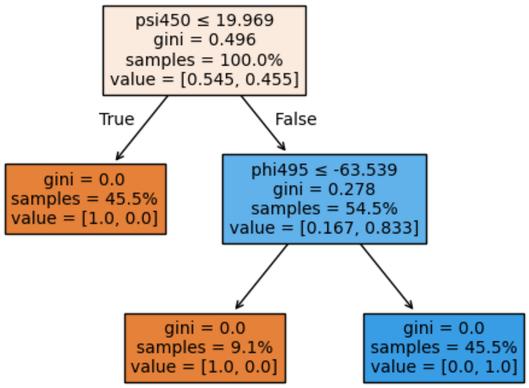

(C)

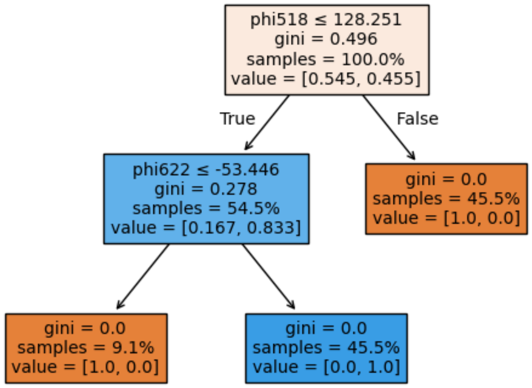

(D)

Figure S3. Decision Trees Used for Feature Selection for Vemurafenib Machine Learning Models Feature selection for vemurafenib using decision trees. (A) The 1st decision tree identified dihedral angles psi622. (B) The 2nd decision tree identified dihedral angles phi484. (C) The 3rd decision tree identified dihedral angles psi450 and phi495. (D) The 4th decision tree identified dihedral angles phi518 and phi622. Each decision tree node shows the decision assessed at that node, its corresponding Gini coefficient, the percentage of samples subjected to this condition, and the repartitioning of those samples into classes. Node colors indicate the most probable class, where orange represents drug sensitive for vemurafenib, blue represents drug resistance for vemurafenib.
